# Infection-related research in Parkinson’s disease: an exploratory trends analysis

**DOI:** 10.3389/fnagi.2026.1959926

**Published:** 2026-10-16

**Authors:** Li-Qin Xiang, Yan-Jun Chen, Sheng-Qiang Zhou, Fang Liu

**Affiliations:** 1 Graduate School of Hunan University of Chinese Medicine, Changsha, China; 2 National TCM Master Liu Zuyi Inheritance Studio, Hunan Provincial Hospital of Integrated Traditional Chinese and Western Medicine (The Affiliated Hospital of Hunan Academy of Traditional Chinese Medicine), Changsha, China

**Keywords:** bibliometrics, infection, neuroinflammation, Parkinson's disease, α-synuclein pathology

## Abstract

**Background:**

Infection and Parkinson's disease (PD) appear to interact through complex mechanisms. Infection may promote the occurrence and progression of pathological processes related to PD, while PD and its associated dysfunctions may increase the risk of infection. This study depicts the evolution, thematic structure, and emerging directions of infection-related research in PD.

**Methods:**

Publications related to infection and PD from 1999 to June 30, 2026 were retrieved from the WoSCC and Scopus databases. R software packages bibliometrix, CiteSpace, and VOSviewer were used for data analysis.

**Results:**

The number of published studies on PD and infection showed a gradual upward trend and increased significantly after 2020. The United States published the largest number of documents (*n* = 509) and received the most citations (*n* = 33,628); China and the UK published 218 and 164 papers, respectively. The University of Florida is the most productive institution (*n* = 23). Dr. Rajesh Pahwa and Dr. Michael S. Okun are among the most productive authors (*n* = 12). Parkinsonism & Related Disorders (*n* = 59) and Movement Disorders (*n* = 46) are the most active journals in this field. The keyword analysis identified five main themes: infection exposure and clinical outcomes; infection-induced neuroinflammation; shared mechanisms of neurodegeneration; virus-related neurological manifestations and PD; and gut microbiota–gut–brain interactions involved in PD pathogenesis and treatment response.

**Conclusion:**

The research on infection and PD has developed rapidly, especially since the COVID-19 epidemic. This field is going beyond epidemiological association to explore how infection can trigger PD through neuroinflammation, α-synuclein (α-syn) pathology, the gut brain axis, and other factors. Infection management in patients with PD has also become an important clinical concern. This study reviews the development of this field, defines the emerging direction, and provides a reference for future mechanism research and clinical practice.

## 1 Introduction

Parkinson’s disease (PD) is the second most common progressive neurodegenerative disorder worldwide. Pathologically, it is mainly characterized by the degeneration of dopaminergic neurons and the deposition of abnormally folded α-synuclein (α-syn) in the brain (Kalia and Lang, 2015). Its clinical manifestations are not limited to typical motor features, including resting tremor, rigidity, bradykinesia, and postural instability, but also include a series of nonmotor disorders, including olfactory dysfunction, constipation, depression, and cognitive decline (Schapira et al., 2017). According to Global Burden of Disease data, about 11.76 million people suffered from PD in 2021. In the same year, about 388,000 people died of PD worldwide, resulting in 7.47 million disability-adjusted life years (DALYs) (Li et al., 2025). The global disease burden is expected to continue increasing, and the number of people affected is expected to increase to 25.2 million by 2050 (Su et al., 2025). Although the etiology and biological processes of PD have not been fully understood, it is generally believed that the disease is caused by the interaction of multiple factors, such as genetic susceptibility, aging-related changes, environmental influences, and inflammatory responses (Nalls et al., 2019; Pang et al., 2019). Environmental factors play an important role in the development of PD (Yuan et al., 2022). According to previous studies, the risk of PD is related to long-term exposure to certain pesticides, trichloroethylene, tetrachloroethylene, and air pollution (Ascherio et al., 2006; Lee et al., 2022). Experimental and mechanistic studies have linked these environmental exposures to mitochondrial dysfunction, oxidative stress, inflammation, α-syn aggregation, and disruption of the blood–brain barrier (Calderón-Garcidueñas et al., 2008; De Miranda et al., 2021). Moreover, environmental exposures in specific regions have also been associated with endemic or atypical parkinsonism, including cycads-derived neurotoxins in the Western Pacific region and heavy metal pollution in industrial areas in northern France (Menšíková et al., 2023). Therefore, clarifying the impact of environmental exposure on the onset and progression of PD remains an important research focus.

Acute and persistent infections can impair neurological function and have been implicated in various neurological and neurodegenerative conditions, including PD, Alzheimer’s disease (AD), multiple sclerosis, and cognitive decline (Kettunen et al., 2024). Infectious agents such as viruses, bacteria, fungi, and parasites may affect the central nervous system through blood transmission, axonal transport, or by inducing systemic and neuroinflammatory reactions (Dando et al., 2014). Increasing evidence suggests that exposure to some infectious disease pathogens, including hepatitis C virus, influenza virus, and severe acute respiratory syndrome coronavirus 2 (SARS-CoV-2), may be associated with increased susceptibility to PD (Cocoros et al., 2021; Wang B. et al., 2023; Wang A. S. et al., 2023; Wang et al., 2020). A population-based cohort study using Taiwan's national health insurance database has found that *Helicobacter pylori* infection was associated with a 2.29-fold higher risk of PD (Huang et al., 2018). Some scholars believe that the impact of infection may depend on the genetic background of the host (Kline et al., 2021). *Leucine-rich repeat kinase 2* (LRRK2), a major gene associated with autosomal dominant PD, is an important example of this interaction. Genetic variants in the *LRRK2* locus may alter immune responses and affect susceptibility to infection and inflammatory conditions (Weindel et al., 2020). Moreover, infection is a major comorbidity that can affect disease progression and prognosis in patients with PD. Autonomic dysfunction, frailty, cognitive decline, and immunosuppression collectively render those people more susceptible to pathogenic infections. A matched cohort study based on the United Kingdom (UK) Biobank data showed that patients with established PD had a significantly higher risk of infection than matched controls, with a fully adjusted hazard ratio of 3.72 (Hu et al., 2023). Most importantly, infectious events may also aggravate the motor and nonmotor symptoms of PD and lead to greater use of hospital services, higher in-hospital mortality, and increased demand for long-term care (Hogg et al., 2022). During the coronavirus disease 2019 (COVID-19) pandemic, a multicenter cohort study recorded a mortality rate of 19.7% in patients with both PD and COVID-19. This result may partly reflect the apparent frailty of patients with advanced disease (Fasano et al., 2020). The broader clinical and economic burden is also considerable. A retrospective study in the UK estimated that hospitalizations of PD patients cost about 907 million pounds over 4four years. The patients in that cohort also had longer hospital stays and were more likely to die during hospitalization (Low et al., 2015). These studies have triggered interest in therapeutic and preventive approaches targeting infection in PD, including antimicrobial therapy, vaccination, and immunomodulatory strategies (Mercado et al., 2024a, 2024b). Although an increasing number of studies have focused on the association between infection and PD, the comprehensive development review, main topics, and emerging research directions in this field still need to be clarified.

Bibliometric analysis can quantitatively evaluate and visually present the evolution of knowledge and structure of a discipline by examining publication trends, cooperation networks, keyword dynamics, and topic clustering. Therefore, we systematically analyzed the literature on infection and PD to delineate the development of this field, outline the mode of scientific cooperation, identify the main themes and their evolution, and highlight the themes and emerging focus areas that have not been fully studied. The results provide a comprehensive overview, which is helpful in clarifying the role of infection in PD and providing references for the formulation of relevant prevention and treatment strategies.

## 2 Methods and materials

### 2.1 Search strategy and data collection

The literature records were mainly obtained from the Web of Science Core Collection (WoSCC), and the Scopus database was used to supplement the data coverage. The search of the two databases was completed on July 12, 2026. The search strategies were as follows: WoSCC: (TI=("Infectious*" OR "Infection*") OR AB=("Infectious*" OR "Infection*") OR AK=("Infectious*" OR "Infection*")) AND (TI=("Parkinson*") OR AB=("Parkinson*") OR AK=("Parkinson*")); Scopus: (TITLE("Infectious*") OR TITLE("Infection*") OR ABS("Infectious*") OR ABS("Infection*") OR AUTHKEY("Infectious*") OR AUTHKEY("Infection*")) AND (TITLE("Parkinson*") OR ABS("Parkinson*") OR AUTHKEY("Parkinson*")). Specific pathogen terms were excluded. Given that the objective of this study was to conduct a thorough review of research examining the link between infection and PD, rather than focusing on particular infectious agents, a broad spectrum of infection-related terms was employed. This method guaranteed the consistency and replicability of the search strategy, while simultaneously reducing the likelihood of selection bias favoring specific pathogens. The inclusion criteria were as follows: (a) original research articles or review articles concerning infection and PD; (b) publication in English; and (c) publication between January 1, 1999, and June 30, 2026. The exclusion criteria were as follows: (a) studies unrelated to infection and PD; (b) publications written in languages other than English; and (c) non-research or non-review articles.

Two researchers independently evaluated titles, abstracts, and full texts based on pre-established inclusion criteria. Any discrepancies were resolved through discussion, and if required, a third researcher was consulted until a consensus was achieved. After that, bibliographic records of the eligible publications were exported separately from WoSCC and Scopus. The resulting datasets were then merged and processed using a Python-based data-processing procedure. Duplicate records were identified using a hierarchical matching strategy: exact DOI matching was applied first, followed, when necessary, by matching normalized article titles together with publication year and first author. Title normalization included conversion to lowercase, removal of punctuation, and normalization of redundant spaces. For duplicate records identified across the two databases, the WoSCC record was retained as the primary record, with missing information supplemented from Scopus (Supplementary Figure S1). The full deduplication script and matching rules are provided in Supplementary data 1.

### 2.2 Data analysis

The standardized datasets were analyzed using the software listed in Table 1 (Aria and Cuccurullo, 2017; Chen, 2004; van Eck and Waltman, 2010). The analysis covers the cooperation network among countries, institutions, and authors; author output rate based on Lotka's law; journal influence; highlight references; and keyword co-occurrence and theme trends. Before analysis, synonyms, abbreviations, plural forms, and spelling variants of keywords were integrated. The detailed software settings and keyword thesaurus are provided in Supplementary data 2 and Supplementary data 3, respectively. The journal was obtained from the Journal Citation Report (JCR) impact factor for 2026.

**Table 1:** Bibliometric software in this study.

Software	Version	Main application in this study	Reference
R package bibliometrix	–	Thematic trend analysis and Lotka’s law fitting	Aria and Cuccurullo (2017)
VOSviewer	1.6.20	Mapping of country, institution, author, and journal collaboration networks and keyword co-occurrence networks	van Eck and Waltman (2010)
CiteSpace	6.4.R1	Detection of citation bursts in references	Chen (2004)

## 3 Results

### 3.1 Publication trends

This bibliometric analysis incorporated 1,708 eligible publications, including 1,263 original research articles and 445 review articles. The annual publication volume of publications in this field showed an overall upward trend from 1999 to 2026 and this developmental trajectory can be split into three phases: a low-growth phase from 1999 to 2011, during which output remained low with minor fluctuations; a gradual-growth phase from 2012 to 2019, with publications increasing from 38 to 71; and a rapid-growth phase beginning in 2020, with output peaking at 174 publications in 2022. Despite a slight decline thereafter, publication output remained high, indicating sustained research interest in this field (Figure 1).

**Figure 1:**
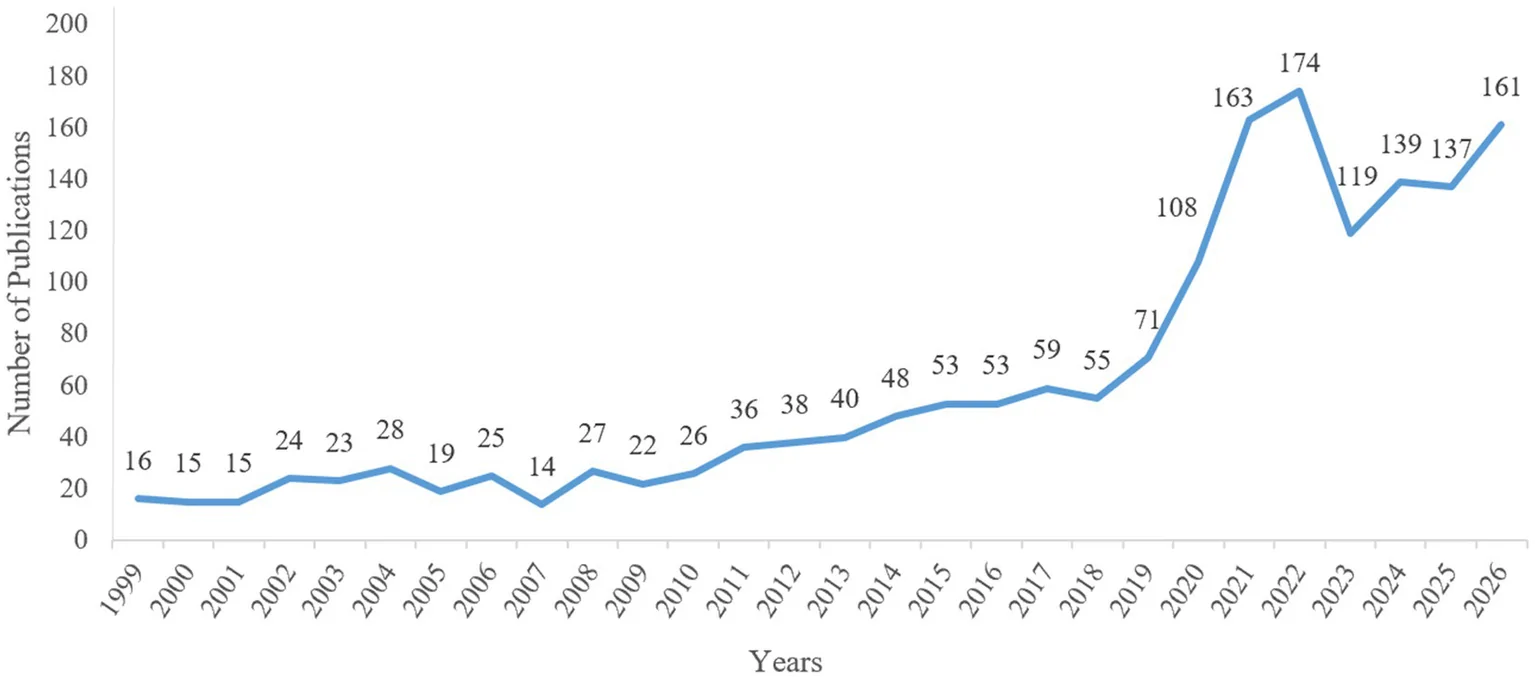
Trends in annual publication output.

### 3.2 Countries

Research on infection and PD was primarily conducted in North America, Europe, and Asia. In terms of research output, the United States (USA) leads with 509 published papers, followed by China with 218. The UK, India, and Italy also demonstrate significant contributions, with 164, 142, and 137 papers published, respectively. The USA also had the highest total number of citations (*n* = 33,628). Despite the relatively low number of studies published in Canada, Germany, and Spain, their articles have each been cited more than 80 times, indicating strong academic impact (Table 2). The international collaboration network spanned multiple continents, with the USA serving as the central hub and maintaining close partnerships with the UK, China, and Germany (Figures 2A,B).

**Figure 2:**
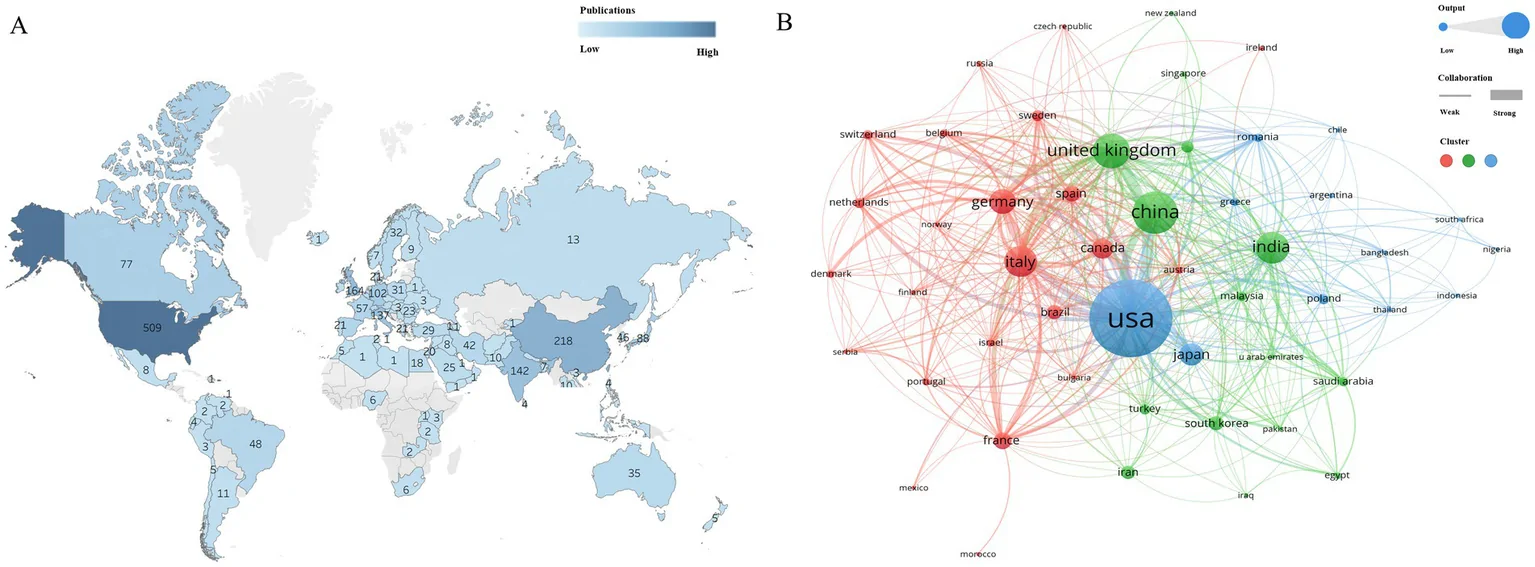
Country analysis. **(A)** Global publication output. **(B)** International collaboration network.

**Table 2:** The top 10 productive countries.

Rank	Country	Documents	Citations	Average citations
1	USA	509	33,628	66.07
2	China	218	5,393	24.74
3	United Kingdom	164	9,516	58.02
4	India	142	3,708	26.11
5	Italy	137	6,514	47.55
6	Germany	102	8,817	86.44
7	Japan	88	2,766	31.43
8	Canada	77	7,488	97.25
9	France	57	3,610	63.33
10	Spain	53	4,468	84.3

### 3.3 Institutions

Institutional cooperation patterns revealed the international research landscape in this field (Figure 3). Among the ten most productive institutions, seven were from the USA, two from the UK, and one from Canada, highlighting the dominance of European and North American institutions. The University of Florida ranked first in publications (*n* = 23), with 5,419 total citations and 235.61 citations per paper. Stanford University ranked second (*n* = 21), with 2,441 citations and 116.24 citations per paper. Columbia University ranked tenth (*n* = 15) but received 2,670 citations, averaging 178.00 citations per paper, indicating strong academic impact (Table 3).

**Figure 3:**
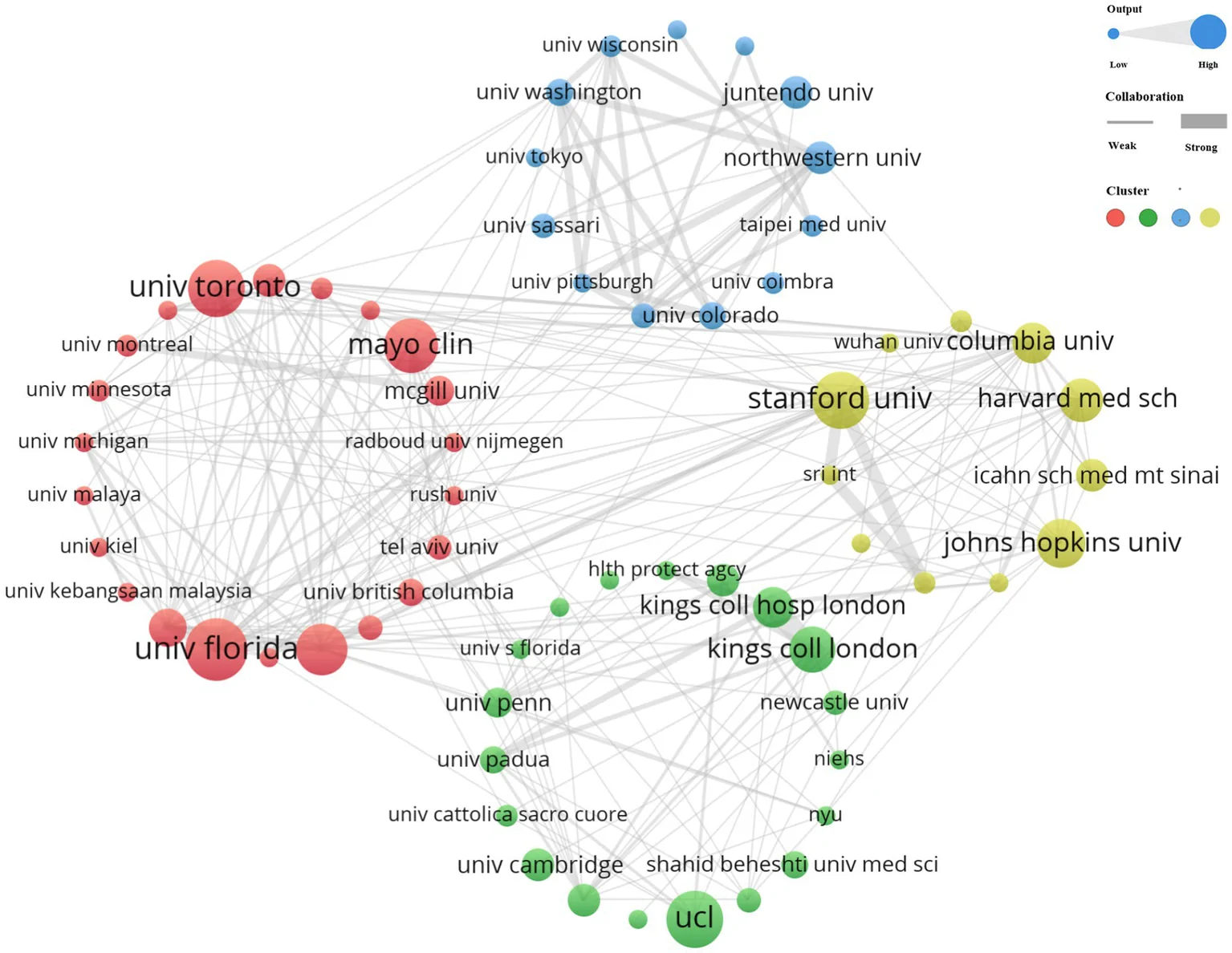
Institutional collaboration network.

**Table 3:** The top 10 productive institutions.

Rank	Organization	Documents	Citations	Average citations	Country
1	University of Florida	23	5,419	235.61	USA
2	Stanford University	21	2,441	116.24	USA
3	University of Toronto	21	2,541	121	Canada
4	University College London	21	538	25.62	UK
5	Mayo Clinic	20	634	31.7	USA
6	University of California	19	2,573	135.42	USA
7	Johns Hopkins University	18	311	17.28	USA
8	King's College London	17	878	51.65	UK
9	Harvard University	16	322	20.13	USA
10	Columbia University	15	2,670	178	USA

### 3.4 Authors

Analysis of high-yield authors revealed that researchers in this field were predominantly from the USA and the UK (Table 4). In accordance with Lotka’s law, the fitted Lotka parameter was *β* = 3.22 (*R*^2^ = 0.9741) (Figure 4A). The classic Lotka model (*β* = 2) differed significantly from the observed distribution (K–S test, *p* = 0.00966), whereas the fitted model showed excellent agreement (*p* = 0.996). The higher *β* value indicated a steeper decline in the frequency of authors as the number of published results increases, highlighting a dominance of low-output authors and a relatively limited size of the core author group with sustained outputs. In the author network, Dr. Rajesh Pahwa from the University of Kansas Medical Center and Dr. Michael S. Okun from the University of Florida both published 12 papers, followed by Dr. Angelo Antonini with 10 papers. These authors are the main researchers in the field. It is worth noting that Dr. Michael S. Okun has been cited for 5,137 times, indicating that his academic research has received widespread attention (Figure 4B).

**Figure 4:**
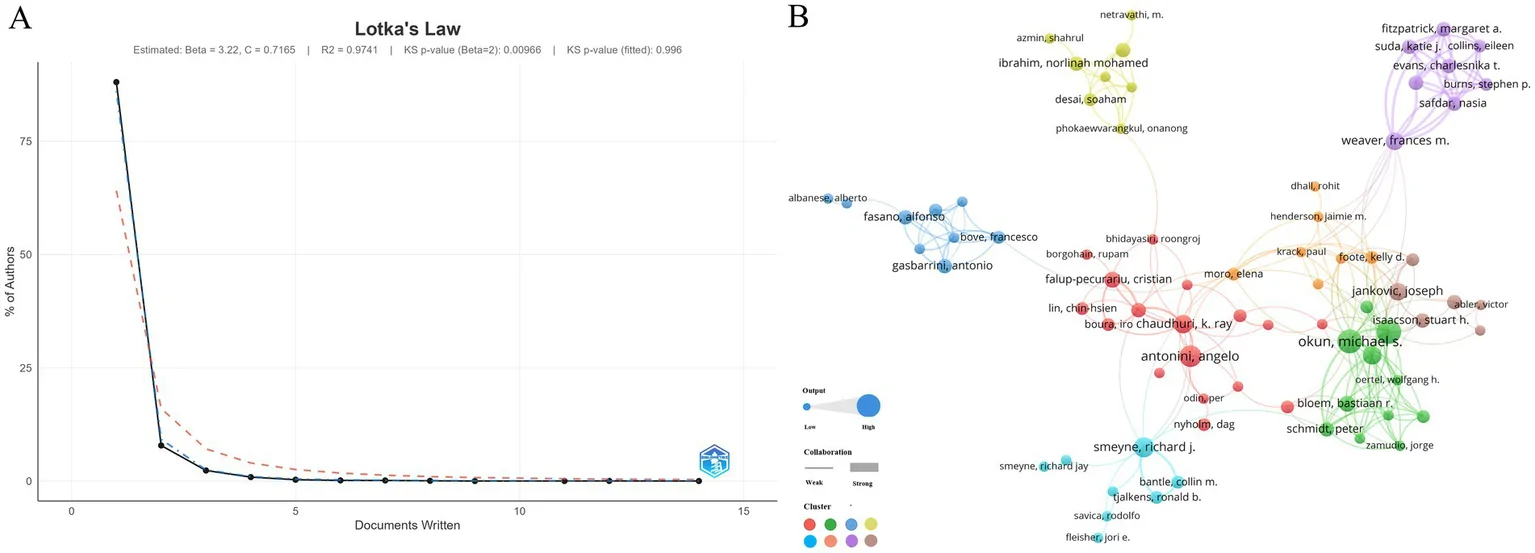
Author analysis. **(A)** Distribution of author productivity based on Lotka’s law; **(B)** Author collaboration network.

**Table 4:** Top 10 productive authors.

Rank	Author	Documents	Citations	Average number of citations	Affiliation	Country
1	Rajesh Pahwa	12	1,677	139.75	University of Kansas Medical Center	USA
2	Michael S. Okun	12	5,137	428.08	University of Florida	USA
3	Angelo Antonini	10	604	60.40	University of Padua	Italy
4	Richard J. Smeyne	9	899	99.89	Thomas Jefferson University	USA
5	Kelly E. Lyons	8	928	116.00	University of Kansas Medical Center	USA
6	K. Ray Chaudhuri	8	582	72.75	King’s College London	UK
7	Patrik Brundin	8	467	58.38	Pharma research and early development	Switzerland
8	Frances M. Weaver	7	895	127.86	Loyola University	USA
9	André Charlett	7	379	54.14	King's College London	UK
10	R. John Dobbs	7	379	54.14	King’s College London	UK

### 3.5 Journals

Journals are key sources of scholarly knowledge. The included articles were published in 762 journals, 86 of which contributed at least four articles (Figure 5A). The most productive journals were *Parkinsonism & Related Disorders* (*n* = 59, IF = 4.3), *Movement Disorders* (*n* = 46, IF = 7.7), *International Journal of Molecular Sciences* (*n* = 31, IF = 5.6), *Journal of Parkinson’s Disease* (*n* = 30, IF = 5.9), and *Frontiers in Neurology* (*n* = 25, IF = 3.3) (Table 5). In the co-citation analysis, 200 journals were cited at least 80 times (Figure 5B). *Movement Disorders* received the most co-citations (*n* = 3,378, IF = 7.7), followed by *Neurology* (*n* = 2,376, IF = 8.9), *Proceedings of the National Academy of Sciences of the United States of America* (*n* = 1,580, IF = 9.5), *PLOS ONE* (*n* = 1,454, IF = 2.8), and *Parkinsonism & Related Disorders* (*n* = 1,338, IF = 4.3) (Table 6).

**Figure 5:**
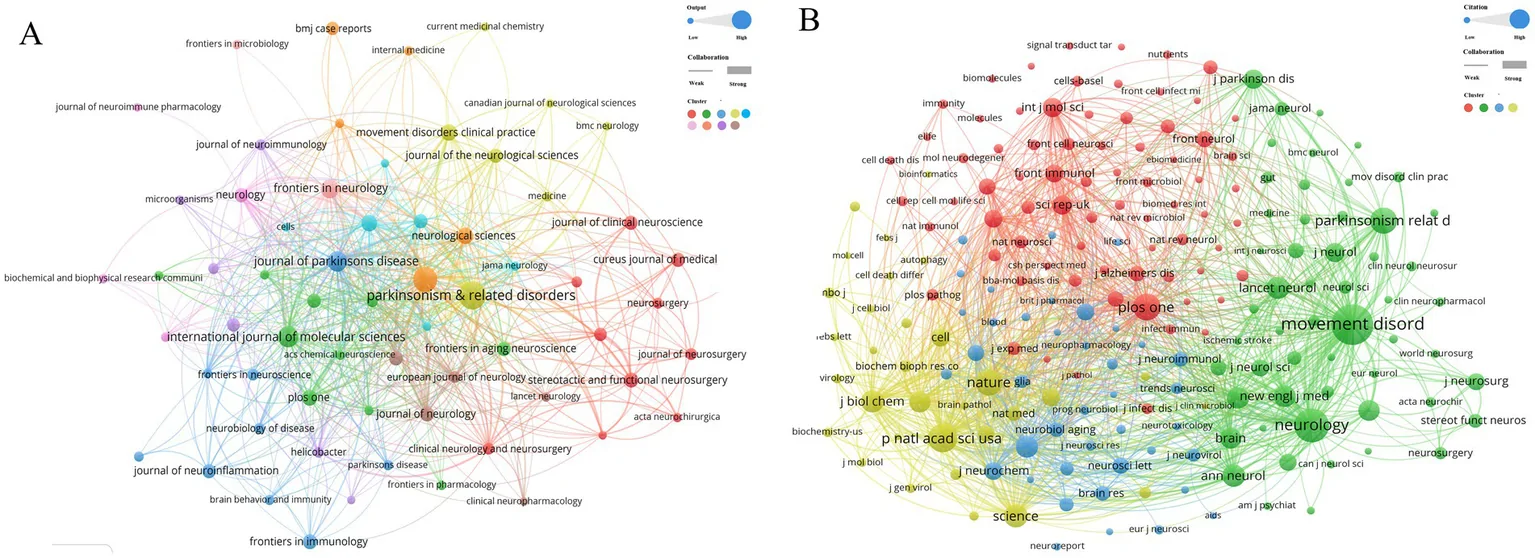
Journal analysis. **(A)** Collaboration network of publishing journals; **(B)** Co-citation network of journals.

**Table 5:** Top 10 active journals.

Rank	Source	Documents	Citations	Average number of citations	IF	JCR
1	Parkinsonism & related disorders	59	2,913	49.37	4.3	Q1
2	Movement disorders	46	3,382	73.52	7.7	Q1
3	International journal of molecular sciences	31	1,179	38.03	5.6	Q1
4	Journal of Parkinsons disease	30	876	29.2	5.9	Q1
5	Frontiers in neurology	25	535	21.4	3.3	Q2
6	Neurological sciences	19	468	24.63	2.7	Q3
7	Journal of neural transmission	18	605	33.61	4.4	Q1
8	Movement disorders clinical practice	18	183	10.17	3.4	Q2
9	PLOS ONE	17	660	38.82	2.8	Q2
10	Stereotactic and functional neurosurgery	16	617	38.56	2.1	Q2

IF, impact factor; JCR, journal citation reports.

**Table 6:** The top 10 co-cited journals.

Rank	Source	Citations	IF	JCR
1	Movement disorders	3,378	7.7	Q1
2	Neurology	2,376	8.9	Q1
3	Proceedings of the National Academy of Sciences of the United States of America	1,580	9.5	Q1
4	PLOS One	1,454	2.8	Q2
5	Parkinsonism & related disorders	1,338	4.3	Q1
6	Nature	1,313	56.1	Q1
7	Science	1,041	47.3	Q1
8	Annals of neurology	981	7.5	Q1
9	Journal of biological chemistry	953	4.1	Q2
10	Lancet neurology	939	54.6	Q1

IF, impact factor; JCR, journal citation reports.

### References

3.6

Citation analysis identified three highly influential studies in infection-related PD research (Figure 6A and Table 7). The most frequently co-cited article, titled *Staging of Brain Pathology Related to Sporadic Parkinson’s Disease* (*n* = 98), introduced the classical Braak staging system. Its findings, which suggest that PD pathology may affect the olfactory system, dorsal motor nucleus of the vagus, and brainstem before the onset of overt symptoms, provide a pathological foundation for the hypothesis that infection may contribute to prodromal neurodegeneration (Braak et al., 2003). The second-ranked article, *Viral Parkinsonism* (*n* = 77), reviewed evidence connecting several viruses—including coxsackievirus, Japanese encephalitis virus, West Nile virus, and HIV—to parkinsonism (Jang et al., 2009a; Jang et al., 2009b). The third influential study, *Highly Pathogenic H5N1 Influenza Virus Can Enter the Central Nervous System and Induce Neuroinflammation and Neurodegeneration* (*n* = 69), demonstrated that the H5N1 could enter the central nervous system via peripheral neural pathways and trigger sustained neuroinflammation (Jang et al., 2009a; Jang et al., 2009b). The authors linked these findings to a “hit-and-run” mechanism, whereby the infection resolves but its secondary pathological effects endure. These studies have played a foundational role in shaping research on infection and PD.

**Figure 6:**
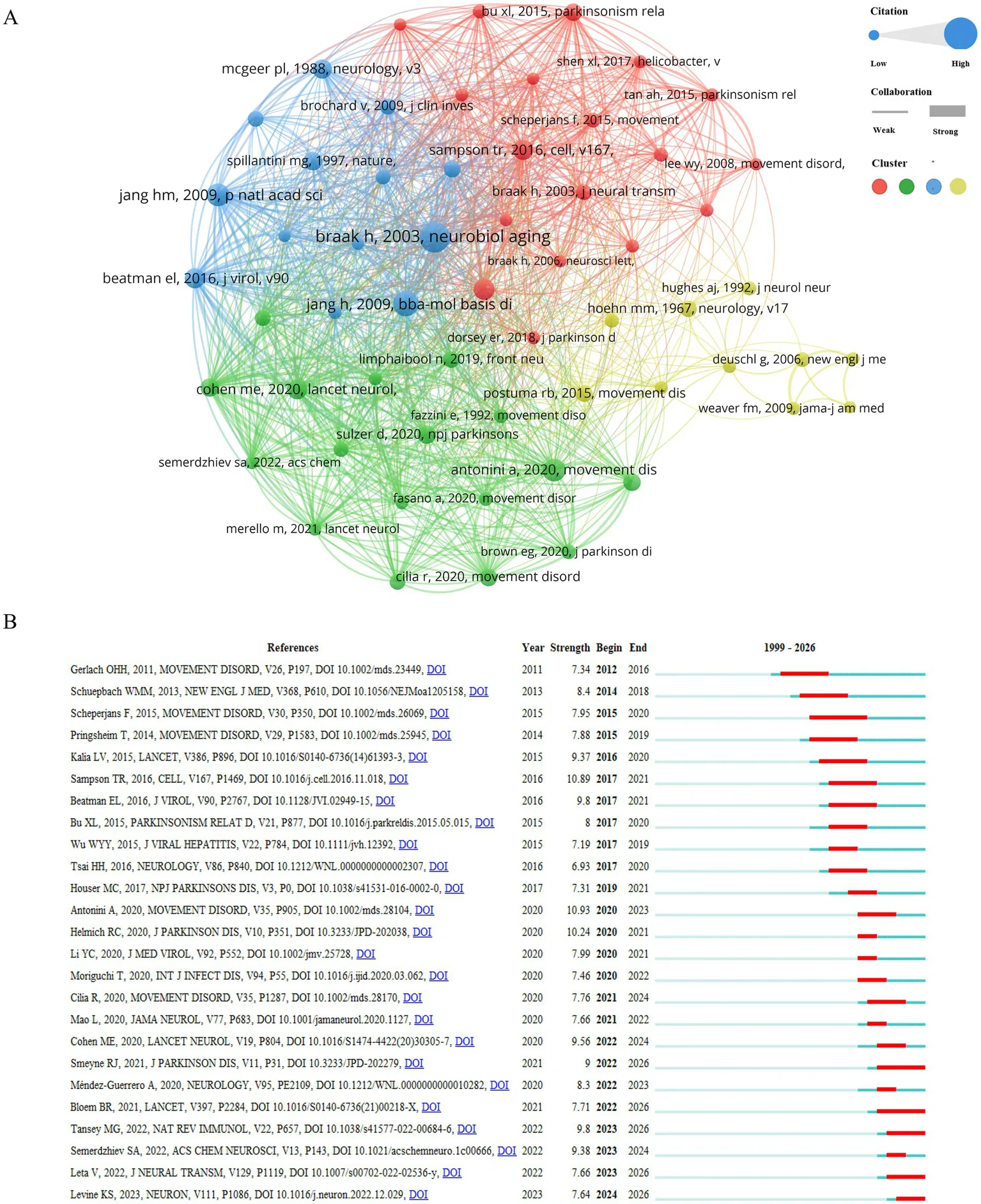
Reference analysis. **(A)** Co-citation network of references; **(B)** Top 25 references with the strongest citation bursts. Red bars indicate the periods of citation bursts, and “Strength” represents burst intensity.

**Table 7:** Top 10 co-cited references.

Rank	Title	Citations	TLS	Year	Journal	IF
1	Staging of brain pathology related to sporadic Parkinson's disease	98	850	2003	Neurobiology of aging	4
2	Viral parkinsonism	77	726	2009	Neurobiology of aging	4
3	Highly pathogenic H5N1 influenza virus can enter the central nervous system and induce neuroinflammation and neurodegeneration	69	823	2009	Proceedings of the National academy of sciences of the United States of America	9.5
4	Outcome of Parkinson's disease patients affected by COVID-19	66	619	2020	Movement disorders	7.7
5	Parkinson's disease	62	486	2015	Lancet	109
6	A case of probable Parkinson's disease after SARS-CoV-2 infection	59	774	2020	Lancet neurology	54.6
7	Lymphatic dissemination of simian immunodeficiency virus after penile inoculation	58	795	2016	Journal of virology	4.1
8	Gut microbiota regulate motor deficits and neuroinflammation in a model of Parkinson's disease	58	605	2016	Cell	45.1
9	Reactive microglia are positive for HLA-DR in the substantia nigra of Parkinson’s and Alzheimer’s disease brains	55	462	1988	Neurology	8.9
10	Acute hypokinetic-rigid syndrome following SARS-CoV-2 infection	52	727	2020	Neurology	8.9

TLS, total link strength; IF, impact factor.

Reference citation bursts reflect shifts in research frontiers and scholarly attention over time. By setting the number of states to 2 and defining a minimum burst duration of 2 years, we identified 154 references with notable citation bursts, with the top 25 highlighted in Figure 6B. The most intense burst was attributed to Antonini et al.’s 2020 article titled *Impact of COVID-19 on Parkinson*’*s Disease* (strength: 10.93; 2020–2023). This was closely followed by Sampson et al.’s (2016) study, *Gut Microbiota Regulate Motor Deficits and Neuroinflammation in a Model of Parkinson*’*s Disease* (strength: 10.89; 2017–2021). Another significant burst was observed for Beatman et al.’s 2016 article, *West Nile Virus Infection Induces α-Synuclein Pathology in Mice* (strength: 9.80; 2017–2021). These prominent citation bursts underscore that COVID-19, gut microbiota, and α-syn pathology have emerged as pivotal topics in recent research on infection and PD. In addition, five references remained in an active burst through 2026, primarily addressing viral exposure and PD risk, neuroinflammation, and COVID-19, suggesting that these topics continue to attract substantial research interest.

### 3.7 Keywords

High-frequency keywords revealed the main research themes in infection and PD (Table 8). After excluding the search terms, the 15 most frequent keywords were AD (*n* = 224), COVID-19 (*n* = 179), α-syn (*n* = 165), deep brain stimulation (DBS, *n* = 144), neuroinflammation (*n* = 126), neurodegeneration (*n* = 121), central nervous system (*n* = 113), neurodegenerative diseases (*n* = 109), risk (*n* = 107), inflammatory responses (*n* = 105), SARS-CoV-2 (*n* = 98), disease (*n* = 98), brain (*n* = 93), complications (*n* = 83), and dementia (*n* = 77). These keywords highlight several major themes, including infection-related neurodegenerative risk, the neurological effects of COVID-19, α-syn pathology, neuroinflammation, and infections associated with DBS. Notably, “AD” was the most frequent keyword, potentially indicating a growing focus on the potential impact of infections in the etiology of neurodegenerative diseases. Both AD and PD exhibit several infection-related mechanisms, including neuroinflammation, microglial activation, and abnormal protein accumulation. This suggests that insights derived from research within the AD domain could provide valuable references for exploring the connection between infection and PD.

**Table 8:** Top 10 active keywords.

Rank	Keywords	Occurrences	TLS
1	AD	224	1317
2	COVID-19	179	905
3	α-syn	165	1057
4	DBS	144	646
5	Neuroinflammation	126	845
6	Neurodegeneration	121	729
7	Central nervous system	113	729
8	Neurodegenerative diseases	109	577
9	Risk	107	636
10	Inflammatory responses	105	671
11	SARS-CoV-2	98	576
12	Disease	98	461
13	Brain	93	532
14	Complications	83	414
15	Dementia	77	430

AD, Alzheimer’s disease; α-syn, α-synuclein; DBS, deep brain stimulation; TLS, total link strength.

Keyword co-occurrence analysis identified five primary thematic clusters, as illustrated in Figure 7. The red cluster, comprising 79 keywords, is centered around terms such as DBS, complications, surgery, mortality, and quality of life. This cluster primarily delves into infectious complications and clinical outcomes associated with the treatment of PD. The green cluster, with 44 keywords, is characterized by terms like α-syn, neurodegenerative diseases, oxidative stress, mitochondrial dysfunction, protein aggregation, and autophagy. It sheds light on the molecular mechanisms that interlink infection and neurodegeneration. The blue cluster, containing 38 keywords, encompasses neuroinflammation, microglia, cytokines, and inflammation, focusing on the immune activation and neuronal damage that infection can trigger. The yellow cluster, made up of 23 keywords, includes COVID-19, SARS-CoV-2, influenza, encephalitis, and post-encephalitic parkinsonism. It covers virus-related neurological symptoms and the manifestations of parkinsonism. Lastly, the purple cluster, with 13 keywords, is represented by Helicobacter pylori, gut microbiota, gut–brain axis, dysbiosis, and levodopa. It underscores the potential influence of gastrointestinal infections and microbial imbalances on the pathogenesis of PD, motor fluctuations, and the bioavailability of levodopa. The complete list of keywords can be found in Supplementary data 4.

**Figure 7:**
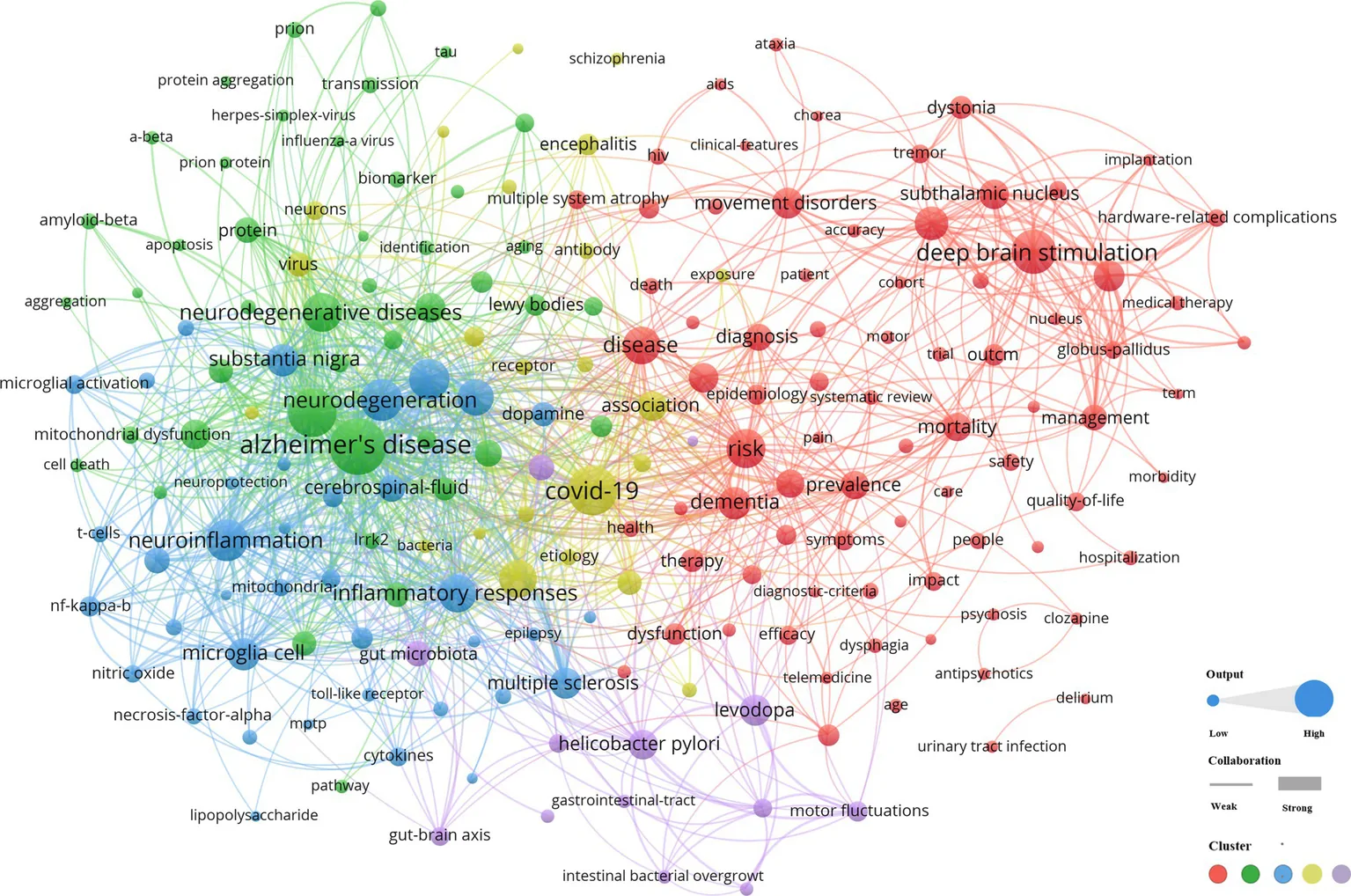
Keyword co-occurrence network.

Trend analysis revealed that studies on infection and PD exhibited distinct temporal evolution patterns (Figure 8). From 2000 to 2010, research efforts were predominantly directed towards specific infectious agents and the foundational pathology of PD, including investigations into AIDS, the Japanese encephalitis virus, Nocardia, the substantia nigra, and dopamine. Between 2011 and 2019, the research landscape expanded to encompass DBS, its associated complications, the subthalamic nuclei, prions, viruses, and parkinsonism. Since 2020, there has been a growing focus on COVID-19, α-syn, and neurodegenerative diseases, while the gut-brain axis, gut microbiota, and neurological diseases have emerged as the latest frontiers in research.

**Figure 8:**
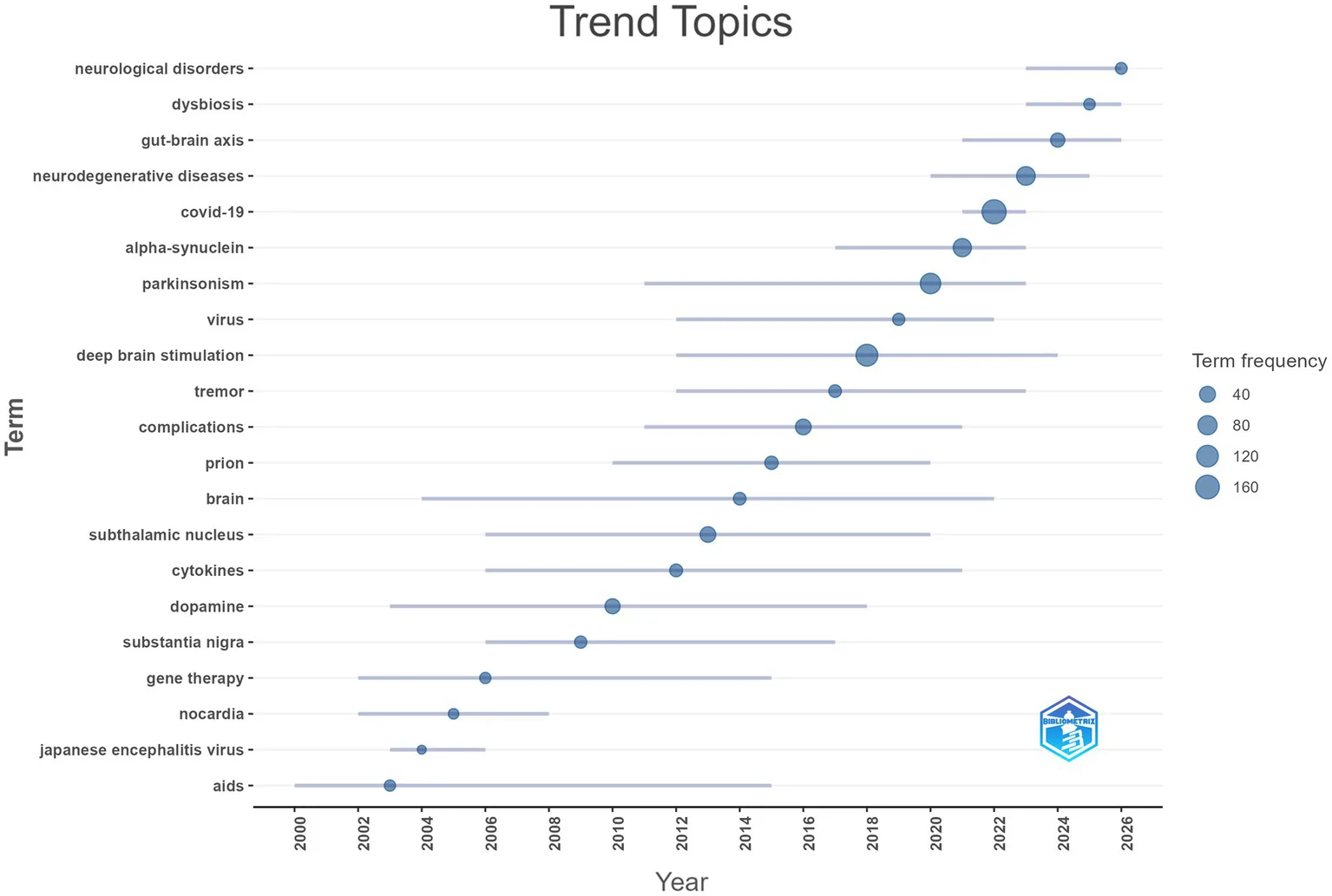
Thematic evolution map. Horizontal lines indicate the active period of each topic, and bubble size represents term frequency.

## 4 Discussion

### 4.1 General information

Between 1999 and 2026, 1,708 publications on infection and PD were identified. Research output has steadily increased over time, especially after 2020. Among contributing countries, the USA showed the highest research productivity and played a prominent role in global collaborative networks, with Canada, Germany, and the United Kingdom also making substantial contributions. Notably, research activities from India and China have expanded considerably in recent years, reflecting the growing involvement of emerging scientific communities. These publications were disseminated across 762 journals and involved 2,972 institutions worldwide. *Parkinsonism & Related Disorders* and *Movement Disorders* represented the major publication venues, whereas the University of Florida and Stanford University showed the highest institutional contributions. These findings indicate that infection-related PD research has evolved into an increasingly collaborative and internationally connected field.

### 4.2 Research hotspots and trends

Combining keyword analysis with reference analysis, we revealed three major themes in the study of infection-related PD: the associations of infectious exposures with PD risk, the mechanistic links between infection and PD pathology, and the clinical burden of infections and their management in patients with PD. These topics are further explored in the following sections (Figure 9).

**Figure 9:**
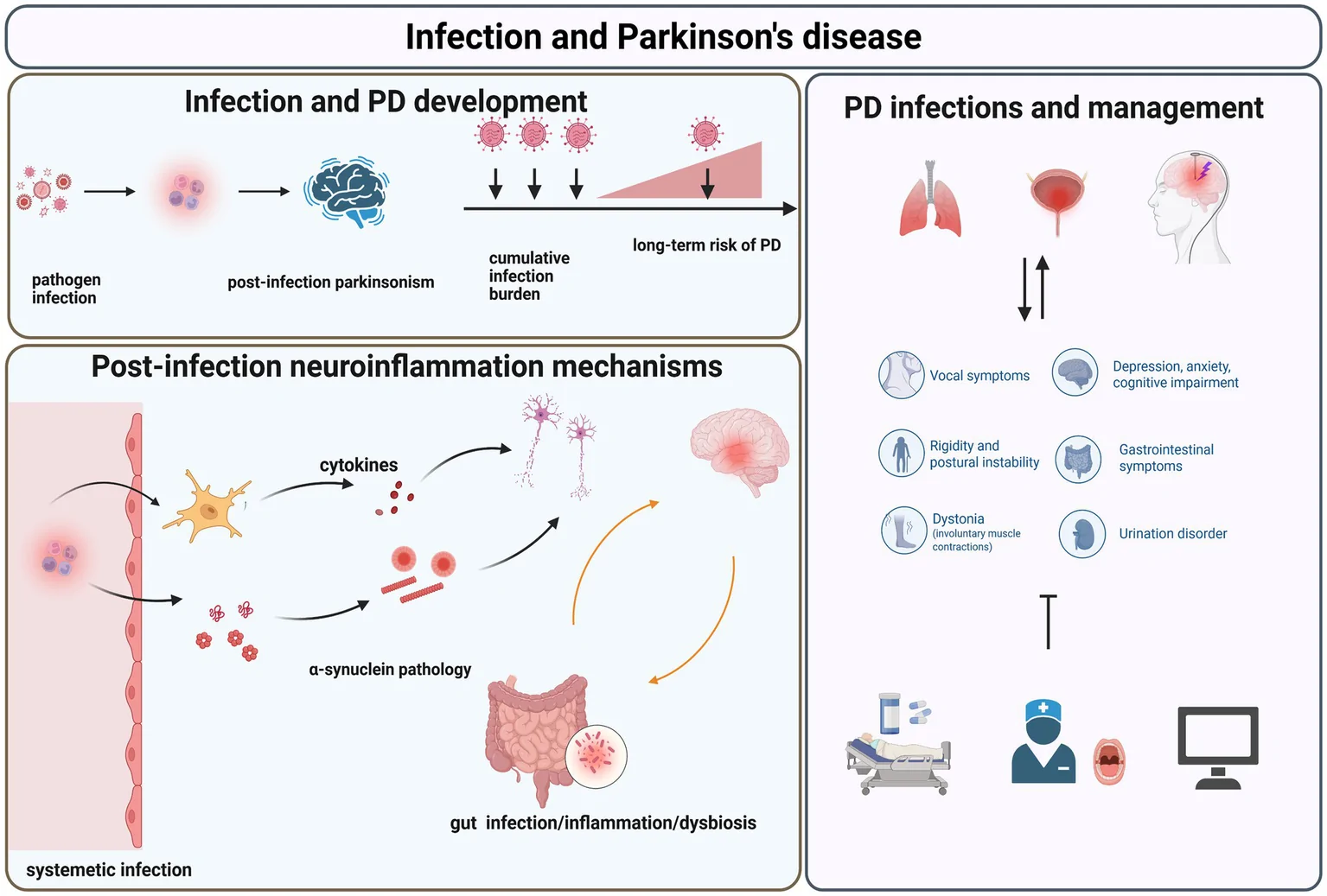
Interplay between infection and Parkinson’s disease. This figure was created with BioRender.com.

#### 4.2.1 Association between infectious exposures and the risk of PD

Research on the association between infection and PD arose from clinical observations. Following the epidemic of encephalitis lethargica in the early twentieth century, many patients developed parkinsonian symptoms (Hoffman and Vilensky, 2017). A variety of neurotropic viral infections can cause acute or subacute parkinsonism, and keyword clusters such as “virus,” “influenza,” and “postencephalitic parkinsonism” reflect this early stage of research. A review summarized evidence linking several viral infections, including Japanese encephalitis virus, West Nile virus, Epstein–Barr virus, herpes simplex virus, and coxsackievirus, to parkinsonism or PD-related neurological manifestations (Jang et al., 2009a; Jang et al., 2009b). H5N1 is a highly pathogenic avian influenza virus, which can cause acute neurological symptoms such as dyskinesia, encephalitis, and coma (Jang et al., 2009a; Jang et al., 2009b). HIV-related parkinsonism has been a focus of previous studies. In some patients, parkinsonism may be an early manifestation of HIV infection and may improve with antiretroviral therapy (Hersh et al., 2001). In untreated patients or those with advanced AIDS, movement disorders may also be associated with HIV-related encephalopathy, severe immunosuppression, opportunistic infections, and exposure to dopamine receptor-blocking drugs (Mirsattari et al., 1998; Tanaka et al., 2000; Tisch and Brew, 2009). Since the outbreak of the COVID-19 pandemic, “COVID-19” and “SARS-CoV-2” have become the core high-frequency terms in this field. Available study shows that COVID-19 can aggravate motor and non-motor symptoms in existing PD patients, and may advance the clinical manifestation onset of individuals at the prodromal stage (Cilia et al., 2020; Leta et al., 2021). Moreover, keywords such as “bacteria” and “toxoplasma gondii” indicate continued efforts to explore potential pathogens. Some bacterial infections are associated with acute parkinsonism and other movement disorders. The immune response induced by infection and the production of anti-basal ganglia antibodies is considered potential underlying mechanisms (Cassarino et al., 2003; Kohbata and Beaman, 1991; McKee and Sussman, 2005; Turkel et al., 2015). Toxoplasma gondii is a common neurotropic parasite, and chronic infection may affect nervous system function; however, a higher-level meta-analysis showed that there was no significant association between Toxoplasma gondii infection and the risk of PD (Bayani et al., 2019). These findings underscore the urgent need for higher-level evidence to clarify whether pathogen exposure contributes to the onset of PD.

Keywords such as “risk,” “burden,” “epidemiology,” and “follow-up” highlight the growing concern about the long-term association between previous infection and PD, especially the potential role of cumulative infection exposure rather than isolated infection events. Bu et al. (2015) reported that patients with PD had a higher cumulative infection burden, accompanied by elevated serum concentrations of inflammatory cytokines and α-syn. Zheng et al.'s (2024) long-term follow-up study based on more than 430,000 participants in the UK Biobank found that the risk of subsequent PD in patients hospitalized due to infection increased significantly, and with the increase in the number of infections, the risk of PD further increased. A sibling control analysis reported that individuals who experienced gastrointestinal or respiratory tract infections between the ages of 21 and 30 had a higher risk of developing PD in the future (Vingeliene et al., 2024). However, in these observational studies of infection and PD, reverse causality is still a major problem: although repeated infections may be associated with an increased risk of subsequent PD, in the long prodromal period of PD, unrecognized symptoms themselves may increase the susceptibility to infection, resulting in an obvious two-way association.

In general, the keyword analysis shows that research on infection and PD has gradually evolved from early studies of infection-related neurological syndromes, including “encephalitis” and “postencephalitic parkinsonism,” to long-term epidemiological investigations focusing on “risk,” “burden,” “epidemiology,” and “follow-up,” and has further expanded to the exploration of associations between emerging infectious diseases, such as COVID-19, and PD. These research hotspots reflect both the attention to etiology and clinical aspects in this field. Because the etiology of sporadic PD is not very clear, studying specific pathogen exposure to PD may promote the development of etiological models of infection and identify potential opportunities for early detection and intervention. Future research should consider pathogen type, exposure intensity, age at the time of infection, and observation window period, and combine long-term follow-up with biomarker assessment in the prodromal phase to clarify the role of infection in the onset and progression of PD.

#### 4.2.2 Potential mechanisms linking infection to PD pathology

Neuroinflammation and its downstream neurodegenerative consequences represent major mechanistic hotspots in this field. The frequent occurrence of keywords “neuroinflammation,” “inflammatory responses,” “microglia cells,” and “cytokines” indicates that microglia-mediated neuroinflammation resulting from infection has become an important research direction. Infection-induced pyroptosis and cytokine release of peripheral myeloid cells can trigger microglial immune training and amplify inflammatory signaling (Wang B. et al., 2023; Wang A. S. et al., 2023). Once activated, microglia recognize pathogen-associated molecular patterns and damage-associated molecular patterns through pattern-recognition receptors, leading to NF-κB activation and increased expression of inflammasome-related components, followed by the release of pro-inflammatory mediators such as IL-1β, IL-6, and TNF-α (Qin et al., 2007; Soraci et al., 2023). In PD, persistence of this inflammatory state can increase the vulnerability of nigrostriatal dopaminergic neurons and contribute to progressive neurodegeneration (Gao et al., 2002).“α-syn” is an important node connecting infection and PD core pathology in the keyword network. In the context of α-syn pathology, such microglial priming may develop into persistent and widespread activation (Mercado et al., 2024a, 2024b), thereby sustaining neuroinflammation and neuronal injury. Furthermore, recent findings imply that α-syn may participate in host antiviral defense through limiting virus replication (Beatman et al., 2015), promoting immune cell recruitment and enhancing neuronal type I interferon signaling (Monogue et al., 2022; Stolzenberg et al., 2017). These findings suggest that infection-induced upregulation of α-syn may initially be protective. When this elevation persists and exceeds the physiological range, it may promote the abnormal aggregation of α-syn and become an important link in infection-induced pathological changes. Moreover, keywords like “oxidative stress,” “autophagy,” “mitochondrial dysfunction,” and “protein aggregation” revealed the potential mechanism of the transformation of infection-related inflammation to neurodegeneration. Persistent inflammation can promote the production of reactive oxygen species and reactive nitrogen radicals, damage the mitochondrial respiratory chain, and interfere with the degradation of abnormal proteins by the autophagy-lysosome system. In this context, α-syn is more prone to abnormal modification, oligomerization, and fibrosis, and the formed aggregates can further activate glial cells and inflammatory pathways, thus forming a vicious cycle of mutual promotion of inflammatory response and protein aggregation. The above process is considered to be an important biological basis for infection exposure that may affect the pathological progression of PD.

In addition, the continued prominence of keywords such as “gut microbiota,” “gut–brain axis,” and “dysbiosis” reflects growing interest in the intestinal contribution to PD. The gut microbiota may contribute to PD progression through immune, vagal, and metabolic pathways (He et al., 2024). Infections can remodel the intestinal flora by triggering systemic inflammation and inducing metabolic changes in the host (Deriu et al., 2016; Groves et al., 2020). This, in turn, weakens the intestinal barrier function, facilitating the entry of microbial products like LPS into the bloodstream, which subsequently intensifies peripheral inflammation and activates central microglia (Forsyth et al., 2011; Qin et al., 2007). Dysbiosis induces alterations in microbial metabolic signals, including short-chain fatty acids and bile acids, consequently influencing neuroinflammation and the homeostasis of α-syn (Li et al., 2021; Sampson et al., 2016). Furthermore, research has demonstrated that intestinal infections can directly trigger pathology associated with PD. Magalhães et al. discovered that Listeria infection can induce mitochondrial dysfunction and damage-associated molecular pattern release, thereby activating innate immune responses and promoting abnormal α-syn aggregation in intestinal and midbrain neurons (Magalhães et al., 2023). Matheoud et al. (2019) found that in Pink1-deficient mice, infection may also trigger mitochondrial antigen presentation and activate cytotoxic CD8 + T cells, leading to axonal injury in dopaminergic neurons. These studies provide emerging evidence for the pathological mode of “intestinal initiation and brain amplification” in PD. Notably, in this research cluster, “*Helicobacter pylori*” is one of the pathogen-related keywords with the highest frequency and is closely associated with keywords such as “eradication” and “levodopa.”. Persistent *Helicobacter pylori* infection can trigger gastric mucosal inflammation and alter the gastric microbiota through virulence factors such as vacuolating toxin A (Radin et al., 2011). Clinical studies have shown that *Helicobacter pylori-*infected people tend to have more severe movement disorders (Tan et al., 2015). Furthermore, *Helicobacter pylori* infection is associated with decreased levodopa response and increased variability in symptom control (Lee et al., 2008). The underlying mechanisms may involve changes in the gastric environment, delayed gastric emptying, duodenal inflammation, and the direct effect between pathogenic bacteria and levodopa (Lee et al., 2008; Niehues and Hensel, 2009; Pierantozzi et al., 2006). Beyond peripheral infections and enteric pathogens, fungal- and bacterial- associated antigens and DNA were detected in postmortem brain tissue of PD, which also provided another kind of evidence for microbial involvement in neuroinflammation (Pisa et al., 2020). However, the presence of pathogen nucleic acid or antigen does not prove the existence of active infection in the brain, and factors such as sampling, preservation, and experimental contamination may also affect the results (Bedarf et al., 2021). Therefore, findings related to brain-associated microbial signatures currently provide only supplementary support for the infection hypothesis.

In general, the results of keyword analysis showed that the mechanism research between infection and PD has formed three interrelated research directions: the neuroimmune response represented by “neuroinflammation,” “microglia cells,” and “cytokines”; the neurodegenerative process represented by “α-syn,” “oxidative stress,” “mitochondrial dysfunction,” “autophagy,” and “protein aggregation”; and the gut-brain axis mechanism represented by “gut microbiota,” “gut-brain axis,” “dysbiosis,” and “*Helicobacter pylori*.” Existing evidence supports that infection-induced persistent inflammation, intestinal microenvironment changes, and host genetic susceptibility are jointly involved in the pathological progression of PD. Still, its specific causality, sequence of action, and key regulatory targets remain to be further clarified.

#### 4.2.3 Clinical burden and management of infections in PD

Infection-related complications, mortality, and clinical management represent another major research focus, as reflected by the frequent co-occurrence of the keywords “complications,” “mortality” and “management.” As a common complication in patients with PD, infection is closely associated with hospitalization, functional decline and elevated mortality risk (Umemura et al., 2014). Following infection, fever, dehydration, and systemic inflammatory reactions can further aggravate motor and non-motor symptoms, forming a vicious cycle of “infection promotes functional deterioration and functional deterioration increases the risk of infection.” Therefore, targeted infection prevention, early recognition, and timely, appropriate treatment should constitute a critical component of the continuum of care for PD. “DBS” is the most frequent keyword in this cluster, reflecting the research priority of infection management related to the surgical treatment of PD. DBS hardware-associated infection is a major complication that compromises surgical efficacy and long-term disease management. Such infections can spread deep along the electrode and extension line, involving the pulse generator or the entire stimulation system. Jia et al. (2024) found that patients who received only local debridement had significantly higher rates of infection recurrence. At the same time removal of all potentially involved hardware reduced the risk of infection spread and recurrence. Long-term follow-up studies further confirm that DBS-related infection is not limited to the early postoperative period, but may run through the whole process of hardware implantation and replacement (Ricciuti et al., 2025). Therefore, the management of DBS-related infections should be stratified according to the anatomical depth of infection and the extent of hardware involvement, to achieve an optimal balance between eradicating the infection and preserving neuromodulation efficacy. Preoperative patient assessment, strict aseptic operation during operation and postoperative incision monitoring are important measures to reduce the incidence of such hardware-related infections the risk of infection (Pritzlaff et al., 2025).

Keywords “pneumonia,” “dysphagia,” “urinary tract infection,” “hospitalization,” and “telemedicine” are important nodes of this cluster. Respiratory tract infection is one of the most common and harmful infections in patients with PD, and dysphagia is the main cause of pneumonia in this population (Patel et al., 2020). Shaibdat et al., (2023) analyzed 605 hospitalization records and found that respiratory infection was the most common reason for emergency admission of PD patients and was independently associated with increased risk of hospitalization complications and death. A retrospective cohort study in South Korea showed that the risk of aspiration pneumonia in patients with PD was significantly higher than that in the general population, and long-term mortality was significantly higher after the first pneumonia (Won et al., 2021). Swallowing function assessment is an important part of long-term follow-up of PD (Patel et al., 2020). It is important for patients with PD to implement oral hygiene management, nutritional support, postural adjustment, and swallowing rehabilitation training (Schindler et al., 2021). Urinary tract infection is another common type of infection in patients with PD, which can lead to acute functional decline. In addition to typical urinary system symptoms including urinary frequency, urgency, and pain, this complication is also closely related to delirium, falls, gait deterioration, and decreased activities of daily living (Hogg et al., 2022). When patients have motor function fluctuations or cognitive deterioration in the short term, priority should be given to the investigation of reversible factors such as infection, dehydration, and medication adherence. The epidemic of COVID-19 has accelerated the application of telemedicine and reshaped the medical service model for patients with PD. Telemedicine can be used for drug adjustment, functional assessment, and rehabilitation guidance, so as to reduce the burden of treatment, especially for elderly patients and patients who need multiple follow-ups after receiving DBS surgery (Lau et al., 2021). In the future, a comprehensive management strategy combining online follow-up and offline evaluation can be considered for patients with PD concurrent infection to achieve better disease control and symptom management. Moreover, antiparkinsonian medications should be used on time and continuously during infection-related hospitalization. Missed or delayed levodopa dosing can rapidly aggravate the symptoms and can induce acute akinesia or parkinsonism-hyperpyrexia syndrome in severe cases (Onofrj and Thomas, 2005; Takubo et al., 2003). For patients who cannot take drugs orally, it is essential that alternative dosing regimens be formulated promptly to maintain the stability of their condition (Safarpour et al., 2026).

Overall, the results of keyword analysis showed that infection was not only a potential disease-related factor in patients with PD, but also an important prognostic factor affecting disease management and clinical outcomes. Studies represented by “DBS,” “complications,” and “management” mainly focus on postoperative infection, while studies represented by “pneumonia,” “urinary tract infection,” “hospitalization,” and “mortality” focus on infection-related complications and adverse outcomes. Whether infection intervention can improve the long-term outcome of PD is an important issue in the field from epidemiological investigation to clinical application. Future research can further carry out standardized infection intervention research for high-risk groups such as DBS treatment, recurrent pneumonia, or urinary tract infection, so as to evaluate the changes in motor symptoms, cognitive function, drug efficacy, and hospitalization risk after the reduction of infection burden.

## 5 Limitations

Several limitations should be acknowledged when considering the findings of this study. Firstly, only English-language publications were included in the study, which may reduce the representativeness of studies published in other languages. However, English is the main language for international scientific exchanges, and the literature included can generally reflect the overall research situation in this field. Secondly, our analysis was confined to WoSCC and Scopus. While these two databases encompass an extensive array of literature and are extensively utilized in bibliometric research, there is a possibility that studies indexed solely in other biomedical or regional databases might be overlooked, potentially resulting in coverage bias. Subsequent research could integrate PubMed or other databases. In addition, subjective screening within the literature review process may introduce some degree of bias in judgments. To mitigate this effect and guarantee uniformity in the selection of studies, we consistently employed pre-established inclusion and exclusion criteria. Finally, bibliometric analysis offers insights into citation, co-occurrence, and clustering patterns within the literature. However, relying solely on these approaches is insufficient to establish a robust biological causal relationship among key themes. To find and confirm their further association and achieve clinical translational, additional evidence is necessary. Nonetheless, the consistency of patterns observed across multiple bibliometric indicators reinforces the overall trends identified in this study. Overall, this research provides a robust foundation for comprehending the progression of this field, elucidating the prevailing research priorities, and pinpointing potential areas for future exploration.

## 6 Conclusion

The research on infection and PD has received extensive attention from scholars all over the world. The USA contributed the largest share of publications and demonstrated substantial academic influence. The University of Florida is the research institution with the highest number of publications, and *Parkinson & related disorders* and *movement disorders* are the most active journals. Infection-related PD research covers epidemiology, pathogenesis, and clinical management. Current epidemiological research focuses on identifying relevant pathogens and linking cumulative infection burden to long-term PD risk. At the same time, mechanistic studies emphasize neuroinflammation, α-syn pathology, and the gut–brain axis. Infection intervention and management are also becoming priorities in PD care. In the future, multi-center, interdisciplinary, and international cooperation should be strengthened, combined with prospective cohort studies, basic mechanism studies, and clinical intervention trials, to further clarify the causal relationship between different infection factors and the occurrence, progression, and clinical phenotype of PD, so as to provide more powerful evidence for the clinical transformation of research in this field.

## Data Availability

The original contributions presented in the study are included in the article/Supplementary material, further inquiries can be directed to the corresponding author.
