# Supplementary material for: Infection-related research in Parkinson’s disease: an exploratory trends analysis
Source: Front Aging Neurosci. 2026 Oct 16;18:1959926. doi: 10.3389/fnagi.2026.1959926 (PMC13725743; doi:10.3389/fnagi.2026.1959926)
Supplement: Supplementary file 1 [file Data_Sheet_1.ZIP › supplementary data 1.docx]

代码网址：[译 英 - 搜索](https://github.com/LeoMengTCM/Bibliometric-Data-Consolidation-Tool/tree/main/src/bibliometrics)

Meeged.py

#!/usr/bin/env python3

\# -*- coding: utf-8 -*-

"""

WOS和Scopus文献数据合并去重工具

策略：

1. WOS记录优先保留（WOS数据更完整）

2. Scopus记录用于补充WOS没有的信息

3. 删除Scopus中与WOS重复的记录

4. 保留Scopus中独有的记录

作者：Meng Linghan

开发工具：Claude Code

日期：2025-11-04

版本：v2.1（优化版）

更新日志：

- 添加logging模块支持

- 改进错误处理和文件验证

- 添加进度显示

"""

import re

import os

import logging

from typing import List, Dict, Set, Tuple

from collections import defaultdict

\# 配置日志

logging.basicConfig(

level=logging.INFO,

format='%(asctime)s - %(levelname)s - %(message)s',

datefmt='%Y-%m-%d %H:%M:%S'

)

logger = logging.getLogger(__name__)

class WOSRecordParser:

"""WOS格式记录解析器"""

@staticmethod

def parse_wos_file(file_path: str) -> List[Dict]:

"""

解析WOS格式文本文件

Returns:

List[Dict]: 记录列表，每条记录是一个字典

"""

with open(file_path, 'r', encoding='utf-8') as f:

content = f.read()

\# 按ER分割记录（ER是记录结束标记）

record_texts = re.split(r'\nER\s*\n', content)

records = []

for record_text in record_texts:

if not record_text.strip() or record_text.strip().startswith('EF'):

continue

\# 解析单条记录

record = WOSRecordParser.parse_single_record(record_text)

if record:

records.append(record)

return records

@staticmethod

def parse_single_record(record_text: str) -> Dict:

"""

解析单条WOS记录

格式：

PT J

AU Author1

Author2

TI Title here

...

"""

record = {}

current_field = None

current_value = []

lines = record_text.split('\n')

for line in lines:

\# 跳过文件头

if line.startswith('FN ') or line.startswith('VR '):

continue

\# 检查是否是新字段（两个字母标签）

field_match = re.match(r'^([A-Z][A-Z0-9])\s+(.*)$', line)

if field_match:

\# 保存前一个字段

if current_field:

record[current_field] = '\n'.join(current_value)

\# 开始新字段

current_field = field_match.group(1)

current_value = [field_match.group(2)]

elif line.startswith('	') and current_field:

\# 续行（3个空格开头）

current_value.append(line.strip())

elif line.strip() == '':

\# 空行，保存当前字段

if current_field:

record[current_field] = '\n'.join(current_value)

current_field = None

current_value = []

\# 保存最后一个字段

if current_field:

record[current_field] = '\n'.join(current_value)

return record

class RecordMatcher:

"""记录匹配器（用于识别重复）"""

@staticmethod

def normalize_title(title: str) -> str:

"""标准化标题（用于匹配）"""

\# 转小写

title = title.lower()

\# 移除标点

title = re.sub(r'[^\w\s]', '', title)

\# 移除多余空格

title = re.sub(r'\s+', ' ', title).strip()

return title

@staticmethod

def get_first_author(record: Dict) -> str:

"""获取第一作者"""

au = record.get('AU', '')

if au:

first_author = au.split('\n')[0].strip()

return first_author.lower()

return ''

@staticmethod

def is_duplicate(record1: Dict, record2: Dict) -> bool:

"""

判断两条记录是否重复

策略：

1. DOI匹配（最准确）

2. 标题 + 年份 + 第一作者

"""

\# 策略1：DOI匹配

doi1 = record1.get('DI', '').strip().lower()

doi2 = record2.get('DI', '').strip().lower()

if doi1 and doi2 and doi1 == doi2:

return True

\# 策略2：标题 + 年份 + 第一作者

title1 = RecordMatcher.normalize_title(record1.get('TI', ''))

title2 = RecordMatcher.normalize_title(record2.get('TI', ''))

if not title1 or not title2:

return False

\# 标题相似度检查

title_similar = (

title1 == title2 or

(len(title1) > 20 and len(title2) > 20 and

(title1 in title2 or title2 in title1))

)

if not title_similar:

return False

\# 年份匹配

year1 = record1.get('PY', '')

year2 = record2.get('PY', '')

if year1 != year2:

return False

\# 第一作者匹配（如果有）

author1 = RecordMatcher.get_first_author(record1)

author2 = RecordMatcher.get_first_author(record2)

if author1 and author2:

return author1 == author2

else:

\# 没有作者信息，仅凭标题+年份判断

return True

class WOSStandardExtractor:

"""从WOS记录中提取标准格式"""

def __init__(self):

self.institutions = {} # {lowercase: original_format}

self.journals = {}	# {lowercase: original_format}

self.countries = {}	# {lowercase: original_format}

self.authors = {}	# {lowercase: original_format}

\# 常见WOS国家名称参考列表（用于辅助验证）

self.common_wos_countries = {

'usa', 'england', 'peoples r china', 'germany', 'france', 'italy',

'spain', 'canada', 'australia', 'japan', 'south korea', 'brazil',

'india', 'russia', 'netherlands', 'switzerland', 'sweden', 'belgium',

'austria', 'denmark', 'norway', 'finland', 'poland', 'portugal',

'greece', 'czech republic', 'hungary', 'ireland', 'israel', 'turkey',

'turkiye', 'new zealand', 'singapore', 'taiwan', 'thailand', 'malaysia',

'indonesia', 'philippines', 'vietnam', 'mexico', 'argentina', 'chile',

'colombia', 'egypt', 'south africa', 'saudi arabia', 'united arab emirates',

'iran', 'pakistan', 'bangladesh', 'nigeria', 'kenya', 'scotland',

'wales', 'northern ireland', 'north ireland'

}

def _is_valid_country(self, text: str) -> bool:

"""验证文本是否像一个国家名称"""

if not text or len(text) < 3: # 太短

return False

text_stripped = text.strip()

text_lower = text_stripped.lower()

\# ========== 快速通过：在常见WOS国家列表中 ==========

if text_lower in self.common_wos_countries:

return True

\# ========== 严格排除：明显不是国家的特征 ==========

\# 1. 长度检查

if len(text_stripped) > 50: # 太长

return False

\# 2. 不应该包含方括号（那是作者标记）

if '[' in text_stripped or ']' in text_stripped:

return False

\# 3. 不应该包含数字（国家名一般不含数字，但邮编会有）

if any(char.isdigit() for char in text_stripped):

return False

\# 4. 特殊符号检查（只允许字母、空格、连字符、撇号、&、点）

allowed_chars = set('abcdefghijklmnopqrstuvwxyzABCDEFGHIJKLMNOPQRSTUVWXYZ -\'&.')

if not all(c in allowed_chars or c.isspace() for c in text_stripped):

return False

\# 5. 首字母应该大写（国家名都是首字母大写）

if not text_stripped[0].isupper():

return False

\# ========== 关键：排除人名格式 ==========

\# 6. 排除 "Name M" 或 "Name A" 格式（典型的人名：姓 + 单个字母）

words = text_stripped.split()

if len(words) == 2:

\# 检查最后一个词是否是单个大写字母（中间名首字母）

last_word = words[-1].strip('.,')

if len(last_word) == 1 and last_word.isupper():

return False # 这是人名！

\# 7. 排除 "Name M." 格式（带点的中间名首字母）

if len(words) == 2 and words[-1].endswith('.') and len(words[-1]) == 2:

return False # 这是人名！

\# 8. 排除州名缩写格式 "AL USA", "CA USA" 等

if len(words) == 2:

first_word = words[0].strip('.,')

\# 如果第一个词是2个大写字母（州名缩写）

if len(first_word) == 2 and first_word.isupper() and words[1].upper() in ['USA']:

return False # 这是州名+国家

\# ========== 排除常见的非国家词 ==========

invalid_words = [

'dept', 'department', 'division', 'center', 'centre', 'lab', 'laboratory',

'institute', 'university', 'college', 'hospital', 'school', 'faculty',

'street', 'road', 'avenue', 'boulevard', 'zip', 'email', 'tel', 'fax',

'phone', 'building', 'floor', 'room', 'suite', 'box', 'district'

]

for word in invalid_words:

if word in text_lower:

return False

\# ========== 积极特征：更可能是国家 ==========

\# 9. 包含 " R " 或 " & " (如 Peoples R China, Trinidad & Tobago)

if ' r ' in text_lower or text_lower.endswith(' r') or ' & ' in text_lower:

return True

\# 10. 多个单词的名称（国家名通常不是单个词）

\# 但要排除只有2个词且最后一个是单字母的情况（已在上面排除）

if len(words) >= 2:

\# 所有单词都不是单个字母

all_words_valid = all(len(w.strip('.,')) > 1 for w in words)

if all_words_valid:

return True

\# 11. 单个词但长度适中（如 Germany, France, Italy）

if len(words) == 1 and 4 <= len(text_stripped) <= 20:

\# 排除常见的城市名（这个比较难，暂时允许通过）

return True

\# 默认不通过

return False

def extract_from_wos_records(self, wos_records: List[Dict]):

"""从WOS记录中提取所有标准格式"""

logger.info("从WOS记录中提取标准格式...")

for record in wos_records:

\# 提取机构名称（C3字段）

if 'C3' in record:

institutions = [inst.strip() for inst in record['C3'].split(';') if inst.strip()]

for inst in institutions:

inst_lower = inst.lower()

if inst_lower not in self.institutions:

self.institutions[inst_lower] = inst

\# 提取期刊名称（SO字段）

if 'SO' in record:

journal = record['SO'].strip()

journal_lower = journal.lower()

if journal_lower not in self.journals:

self.journals[journal_lower] = journal

\# 提取国家名称（从C1字段）

if 'C1' in record:

\# C1格式: [Author] Institution, City, Country.

\# 注意：必须以句点结尾，且国家名是最后一个逗号后、句点前的内容

lines = record['C1'].split('\n')

for line in lines:

\# 必须包含句点（标准WOS格式以句点结尾）

if '.' in line and ',' in line:

\# 提取句点前的内容

before_period = line.split('.')[0]

\# 再按逗号分割

parts = before_period.split(',')

if len(parts) >= 2: # 至少有机构和国家

country = parts[-1].strip()

\# 验证是否像一个国家名称

if self._is_valid_country(country):

country_lower = country.lower()

if country_lower not in self.countries:

self.countries[country_lower] = country

\# 提取作者名称（AU字段）

if 'AU' in record:

authors = record['AU'].split('\n')

for author in authors:

author = author.strip()

if author:

author_lower = author.lower()

if author_lower not in self.authors:

self.authors[author_lower] = author

logger.info(f"✓ 提取完成：")

logger.info(f" - 机构: {len(self.institutions)} 个")

logger.info(f" - 期刊: {len(self.journals)} 个")

logger.info(f" - 国家: {len(self.countries)} 个")

logger.info(f" - 作者: {len(self.authors)} 个")

\# 显示提取到的国家列表（前20个）

if self.countries:

logger.info(f" 提取到的国家（前20个）:")

for i, country in enumerate(sorted(self.countries.values())[:20], 1):

logger.info(f"	{i}. {country}")

if len(self.countries) > 20:

logger.info(f"	... 还有 {len(self.countries) - 20} 个国家")

def standardize_scopus_record(self, scopus_record: Dict) -> Dict:

"""将Scopus记录标准化为WOS格式"""

standardized = scopus_record.copy()

\# 1. 标准化机构名称（C3字段）

if 'C3' in standardized:

institutions = [inst.strip() for inst in standardized['C3'].split(';') if inst.strip()]

standardized_institutions = []

for inst in institutions:

inst_lower = inst.lower()

\# 如果在WOS中出现过，使用WOS的格式

if inst_lower in self.institutions:

standardized_institutions.append(self.institutions[inst_lower])

else:

standardized_institutions.append(inst)

standardized['C3'] = '; '.join(standardized_institutions)

\# 2. 标准化期刊名称（SO字段）

if 'SO' in standardized:

journal = standardized['SO'].strip()

journal_lower = journal.lower()

if journal_lower in self.journals:

standardized['SO'] = self.journals[journal_lower]

\# 3. 标准化国家名称（C1字段）

if 'C1' in standardized:

lines = standardized['C1'].split('\n')

standardized_lines = []

for line in lines:

\# 必须包含句点和逗号（标准WOS格式）

if '.' in line and ',' in line:

\# 提取句点前的内容

before_period = line.split('.')[0]

after_period = '.'.join(line.split('.')[1:]) # 保留句点后的内容

\# 按逗号分割

parts = before_period.split(',')

if len(parts) >= 2:

country = parts[-1].strip()

\# 如果这是一个有效的国家名且在WOS中出现过，使用WOS的格式

if self._is_valid_country(country):

country_lower = country.lower()

if country_lower in self.countries:

\# 替换为WOS标准格式

parts[-1] = ' ' + self.countries[country_lower]

standardized_line = ','.join(parts) + '.'

if after_period:

standardized_line += after_period

standardized_lines.append(standardized_line)

else:

standardized_lines.append(line)

else:

\# 不是有效的国家名，保持原样

standardized_lines.append(line)

else:

standardized_lines.append(line)

else:

standardized_lines.append(line)

standardized['C1'] = '\n'.join(standardized_lines)

\# 4. 标准化作者名称（AU字段）

if 'AU' in standardized:

authors = standardized['AU'].split('\n')

standardized_authors = []

for author in authors:

author = author.strip()

if author:

author_lower = author.lower()

\# 如果在WOS中出现过，使用WOS的格式

if author_lower in self.authors:

standardized_authors.append(self.authors[author_lower])

else:

standardized_authors.append(author)

standardized['AU'] = '\n'.join(standardized_authors)

\# AF字段（全名）也需要对齐

if 'AF' in standardized:

af_authors = standardized['AF'].split('\n')

if len(af_authors) == len(standardized_authors):

\# 保持AF和AU的顺序一致

standardized['AF'] = '\n'.join(af_authors)

return standardized

class RecordMerger:

"""记录合并器（将Scopus信息补充到WOS）"""

@staticmethod

def merge_scopus_to_wos(wos_record: Dict, scopus_record: Dict) -> Dict:

"""

将Scopus信息补充到WOS记录

Args:

wos_record: WOS记录（优先保留）

scopus_record: Scopus记录（补充信息）

Returns:

Dict: 合并后的记录（以WOS为主）

"""

merged = wos_record.copy()

\# 1. TC（被引次数）：取最大值

tc_wos = int(wos_record.get('TC', '0') or '0')

tc_scopus = int(scopus_record.get('TC', '0') or '0')

if tc_scopus > tc_wos:

merged['TC'] = str(tc_scopus)

merged['Z9'] = str(tc_scopus)

\# 2. 补充缺失字段（从Scopus）

\# 注意：C1和C3（机构信息）必须优先使用WOS的，绝不从Scopus补充！

supplement_fields = ['AB', 'LA', 'DT', 'PU', 'SN', 'J9', 'JI', 'PM', 'DI']

for field in supplement_fields:

if not merged.get(field) and scopus_record.get(field):

merged[field] = scopus_record[field]

\# 3. 机构信息（C1, C3）严格以WOS为准，只在WOS完全缺失时才补充Scopus

\# 这确保机构名称格式始终使用WOS标准

if not merged.get('C1') and scopus_record.get('C1'):

merged['C1'] = scopus_record['C1']

if not merged.get('C3') and scopus_record.get('C3'):

merged['C3'] = scopus_record['C3']

\# 4. 添加来源标记（用于清洗时识别）

merged['_SOURCE'] = 'WOS' # 标记此记录以WOS为基础

return merged

class MergeDeduplicateTool:

"""合并去重工具主类"""

def __init__(self, wos_file: str, scopus_file: str, output_file: str):

"""

初始化合并去重工具

Args:

wos_file: WOS文件路径

scopus_file: Scopus转换后的文件路径

output_file: 输出文件路径

Raises:

FileNotFoundError: 输入文件不存在

"""

\# 文件验证

if not os.path.exists(wos_file):

raise FileNotFoundError(f"WOS文件不存在: {wos_file}")

if not os.path.exists(scopus_file):

raise FileNotFoundError(f"Scopus文件不存在: {scopus_file}")

self.wos_file = wos_file

self.scopus_file = scopus_file

self.output_file = output_file

self.parser = WOSRecordParser()

self.matcher = RecordMatcher()

self.merger = RecordMerger()

self.wos_standard_extractor = WOSStandardExtractor() # WOS标准提取器

self.wos_records = []

self.scopus_records = []

self.final_records = []

self.stats = {

'wos_count': 0,

'scopus_count': 0,

'scopus_duplicates': 0,

'scopus_unique': 0,

'final_count': 0,

'duplicate_details': [],

'yearly_stats': {}, # {year: {'documents': count, 'citations': total}}

'standardized_count': 0 # 标准化的Scopus记录数

}

logger.info(f"初始化合并工具 - WOS: {wos_file}, Scopus: {scopus_file}")

def run(self):

"""执行合并去重流程"""

logger.info("=" * 60)

logger.info("WOS + Scopus 文献合并去重工具 v2.1")

logger.info("=" * 60)

logger.info(f"WOS文件: {self.wos_file}")

logger.info(f"Scopus文件: {self.scopus_file}")

logger.info(f"输出文件: {self.output_file}")

logger.info("")

\# 步骤1：读取文件

logger.info("步骤 1/5: 读取文件...")

self.wos_records = self.parser.parse_wos_file(self.wos_file)

self.scopus_records = self.parser.parse_wos_file(self.scopus_file)

self.stats['wos_count'] = len(self.wos_records)

self.stats['scopus_count'] = len(self.scopus_records)

logger.info(f" 读取WOS记录: {self.stats['wos_count']} 条")

logger.info(f" 读取Scopus记录: {self.stats['scopus_count']} 条")

logger.info("")

\# 步骤2：从WOS记录中提取标准格式

logger.info("步骤 2/5: 从WOS记录中提取标准格式（机构、期刊、国家、作者）...")

self.wos_standard_extractor.extract_from_wos_records(self.wos_records)

logger.info("")

\# 步骤3：识别Scopus中与WOS重复的记录

logger.info("步骤 3/5: 识别WOS-Scopus重复记录...")

wos_scopus_pairs = self.find_wos_scopus_duplicates()

self.stats['scopus_duplicates'] = len(wos_scopus_pairs)

self.stats['scopus_unique'] = self.stats['scopus_count'] - self.stats['scopus_duplicates']

logger.info(f" 发现WOS-Scopus重复: {self.stats['scopus_duplicates']} 条")

logger.info(f" Scopus独有记录: {self.stats['scopus_unique']} 条")

logger.info("")

\# 步骤4：合并记录（WOS优先，Scopus独有记录对齐WOS标准）

logger.info("步骤 4/5: 合并记录（WOS优先，Scopus独有记录对齐WOS标准）...")

self.merge_records(wos_scopus_pairs)

self.stats['final_count'] = len(self.final_records)

logger.info(f" 最终记录数: {self.stats['final_count']} 条")

logger.info(f"	- WOS记录（合并后）: {self.stats['wos_count']} 条")

logger.info(f"	- Scopus独有记录: {self.stats['scopus_unique']} 条")

logger.info(f"	- Scopus记录标准化: {self.stats['standardized_count']} 条")

logger.info("")

\# 步骤5：写入文件

logger.info("步骤 5/5: 写入输出文件...")

self.write_output()

logger.info(f" 输出文件已保存: {self.output_file}")

logger.info("")

\# 步骤5：生成年度统计

logger.info("生成年度统计...")

self.calculate_yearly_stats()

logger.info("")

\# 打印统计报告

self.print_report()

def find_wos_scopus_duplicates(self) -> List[Tuple[int, int]]:

"""

查找WOS和Scopus之间的重复记录

Returns:

List[Tuple[int, int]]: (WOS索引, Scopus索引) 对列表

"""

pairs = []

scopus_matched = set() # 已匹配的Scopus索引

for wos_idx, wos_record in enumerate(self.wos_records):

for scopus_idx, scopus_record in enumerate(self.scopus_records):

if scopus_idx in scopus_matched:

continue

if self.matcher.is_duplicate(wos_record, scopus_record):

pairs.append((wos_idx, scopus_idx))

scopus_matched.add(scopus_idx)

\# 记录详情

title = wos_record.get('TI', 'N/A')[:60]

self.stats['duplicate_details'].append({

'title': title,

'wos_idx': wos_idx,

'scopus_idx': scopus_idx

})

break # 找到匹配，继续下一个WOS记录

return pairs

def merge_records(self, wos_scopus_pairs: List[Tuple[int, int]]):

"""

合并记录

策略：

1. 将WOS-Scopus对合并（Scopus补充WOS）

2. 添加Scopus独有记录，并对齐WOS标准格式

"""

scopus_used = set(scopus_idx for _, scopus_idx in wos_scopus_pairs)

\# 1. 处理WOS记录（合并Scopus信息）

for wos_idx, wos_record in enumerate(self.wos_records):

\# 查找对应的Scopus记录

scopus_record = None

for wos_i, scopus_i in wos_scopus_pairs:

if wos_i == wos_idx:

scopus_record = self.scopus_records[scopus_i]

break

if scopus_record:

\# 合并（以WOS为准）

merged = self.merger.merge_scopus_to_wos(wos_record, scopus_record)

self.final_records.append(merged)

else:

\# WOS独有记录，标记来源

wos_record['_SOURCE'] = 'WOS'

self.final_records.append(wos_record)

\# 2. 添加Scopus独有记录（对齐WOS标准格式）

for scopus_idx, scopus_record in enumerate(self.scopus_records):

if scopus_idx not in scopus_used:

\# ⭐ 关键：对齐WOS标准格式（机构、期刊、国家、作者）

standardized = self.wos_standard_extractor.standardize_scopus_record(scopus_record)

standardized['_SOURCE'] = 'SCOPUS'

self.final_records.append(standardized)

self.stats['standardized_count'] += 1

def write_output(self):

"""写入合并后的WOS格式文件"""

lines = []

\# 文件头（使用标准WOS格式，确保VOSviewer兼容性）

lines.append("FN Clarivate Analytics Web of Science")

lines.append("VR 1.0")

\# 写入每条记录

for i, record in enumerate(self.final_records):

\# 在记录前添加空行（第一条除外）

if i > 0:

lines.append("")

\# PT - Publication Type

lines.append(f"PT {record.get('PT', 'J')}")

\# 移除内部标记字段

record_clean = {k: v for k, v in record.items() if not k.startswith('_')}

\# 所有其他字段（按常见顺序）

field_order = [

'AU', 'AF', 'TI', 'SO', 'LA', 'DT', 'DE', 'ID', 'AB',

'C1', 'C3', 'RP', 'EM', 'RI', 'OI', 'CR', 'NR',

'TC', 'Z9', 'U1', 'U2', 'PU', 'SN', 'EI', 'J9', 'JI',

'PY', 'VL', 'IS', 'SI', 'BP', 'EP', 'AR', 'PG',

'DI', 'WE', 'UT', 'PM', 'DA'

]

for field in field_order:

if field in record_clean and field != 'PT':

value = record_clean[field]

if '\n' in value:

\# 多行字段

value_lines = value.split('\n')

lines.append(f"{field} {value_lines[0]}")

for line in value_lines[1:]:

lines.append(f"	{line}")

else:

\# 单行字段

lines.append(f"{field} {value}")

\# ER - End of Record

lines.append("ER")

\# 空行 + EF

lines.append("")

lines.append("EF")

\# 写入文件（包含UTF-8 BOM，与WOS格式完全一致）

with open(self.output_file, 'w', encoding='utf-8-sig') as f:

f.write('\n'.join(lines))

def calculate_yearly_stats(self):

"""

计算每年的发文量和引文量

统计维度：

- Year: 年份

- Documents: 文档数量

- Citations: 引文总数

"""

yearly_data = defaultdict(lambda: {'documents': 0, 'citations': 0})

for record in self.final_records:

\# 获取年份

year = record.get('PY', '').strip()

if not year:

year = 'Unknown'

\# 获取引文数

citations = record.get('TC', '0').strip()

try:

citations = int(citations) if citations else 0

except ValueError:

citations = 0

\# 累加统计

yearly_data[year]['documents'] += 1

yearly_data[year]['citations'] += citations

\# 排序（按年份）

self.stats['yearly_stats'] = dict(sorted(yearly_data.items()))

logger.info(f" 年度统计完成，覆盖 {len(self.stats['yearly_stats'])} 个年份")

def print_report(self):

"""打印去重报告"""

logger.info("=" * 60)

logger.info("合并去重报告")

logger.info("=" * 60)

logger.info(f"WOS原始记录数: {self.stats['wos_count']} 条")

logger.info(f"Scopus原始记录数: {self.stats['scopus_count']} 条")

logger.info(f"")

logger.info(f"WOS-Scopus重复记录: {self.stats['scopus_duplicates']} 条（已从Scopus删除）")

logger.info(f"Scopus独有记录: {self.stats['scopus_unique']} 条（已保留）")

logger.info(f" ⭐ Scopus独有记录标准化: {self.stats['standardized_count']} 条")

logger.info(f"	（机构、期刊、国家、作者已对齐WOS格式）")

logger.info(f"")

logger.info(f"最终记录数: {self.stats['final_count']} 条")

logger.info(f" = WOS记录（含补充）: {self.stats['wos_count']} 条")

logger.info(f" + Scopus独有: {self.stats['scopus_unique']} 条")

logger.info("")

if self.stats['duplicate_details']:

logger.info("WOS-Scopus重复记录详情（前10条）:")

logger.info("-" * 60)

for i, detail in enumerate(self.stats['duplicate_details'][:10], 1):

logger.info(f"{i}. {detail['title']}...")

logger.info(f"	WOS索引: {detail['wos_idx']}, Scopus索引: {detail['scopus_idx']}")

if len(self.stats['duplicate_details']) > 10:

logger.info(f"... 还有 {len(self.stats['duplicate_details']) - 10} 条重复记录")

\# 年度统计表格

if self.stats['yearly_stats']:

logger.info("")

logger.info("=" * 60)

logger.info("年度发文量及引文量统计")

logger.info("=" * 60)

logger.info(f"{'Year':<10} {'Documents':<15} {'Citations':<15}")

logger.info("-" * 60)

total_docs = 0

total_cites = 0

for year in sorted(self.stats['yearly_stats'].keys()):

data = self.stats['yearly_stats'][year]

docs = data['documents']

cites = data['citations']

total_docs += docs

total_cites += cites

logger.info(f"{year:<10} {docs:<15} {cites:<15}")

logger.info("-" * 60)

logger.info(f"{'Total':<10} {total_docs:<15} {total_cites:<15}")

logger.info("")

logger.info("=" * 60)

logger.info("说明：")

logger.info("- WOS记录优先保留（数据更完整）")

logger.info("- Scopus信息用于补充WOS缺失字段")

logger.info("- 被引次数（TC）取两者最大值")

logger.info("- Scopus独有记录（无WOS对应）已全部保留")

logger.info("- ⭐ Scopus独有记录已对齐WOS标准格式：")

logger.info("	· 机构名称：如果在WOS中出现过，使用WOS格式")

logger.info("	· 期刊名称：如果在WOS中出现过，使用WOS格式")

logger.info("	· 国家名称：如果在WOS中出现过，使用WOS格式")

logger.info("	· 作者名称：如果在WOS中出现过，使用WOS格式")

logger.info("=" * 60)

logger.info("合并去重完成！")

logger.info("=" * 60)

\# 保存详细报告

report_file = self.output_file.replace('.txt', '_report.txt')

with open(report_file, 'w', encoding='utf-8') as f:

f.write("=" * 60 + "\n")

f.write("WOS + Scopus 合并去重详细报告\n")

f.write("=" * 60 + "\n\n")

f.write(f"WOS原始记录数: {self.stats['wos_count']} 条\n")

f.write(f"Scopus原始记录数: {self.stats['scopus_count']} 条\n\n")

f.write(f"WOS-Scopus重复记录: {self.stats['scopus_duplicates']} 条\n")

f.write(f"Scopus独有记录: {self.stats['scopus_unique']} 条\n")

f.write(f" ⭐ Scopus独有记录标准化: {self.stats['standardized_count']} 条\n")

f.write(f"	（机构、期刊、国家、作者已对齐WOS格式）\n\n")

f.write(f"最终记录数: {self.stats['final_count']} 条\n\n")

f.write("=" * 60 + "\n")

f.write("WOS格式对齐说明\n")

f.write("=" * 60 + "\n")

f.write("Scopus独有记录已自动对齐WOS标准格式：\n")

f.write("- 机构名称（C3字段）：如果在WOS中出现过，使用WOS格式\n")

f.write("- 期刊名称（SO字段）：如果在WOS中出现过，使用WOS格式\n")

f.write("- 国家名称（C1字段）：如果在WOS中出现过，使用WOS格式\n")

f.write("- 作者名称（AU字段）：如果在WOS中出现过，使用WOS格式\n\n")

f.write("这确保了所有记录的格式一致性，提高了数据质量和分析准确性。\n\n")

if self.stats['duplicate_details']:

f.write("WOS-Scopus重复记录详情:\n")

f.write("-" * 60 + "\n")

for i, detail in enumerate(self.stats['duplicate_details'], 1):

f.write(f"{i}. {detail['title']}...\n")

f.write(f"	WOS索引: {detail['wos_idx']}, Scopus索引: {detail['scopus_idx']}\n")

\# 年度统计表格

if self.stats['yearly_stats']:

f.write("\n" + "=" * 60 + "\n")

f.write("年度发文量及引文量统计\n")

f.write("=" * 60 + "\n")

f.write(f"{'Year':<10} {'Documents':<15} {'Citations':<15}\n")

f.write("-" * 60 + "\n")

total_docs = 0

total_cites = 0

for year in sorted(self.stats['yearly_stats'].keys()):

data = self.stats['yearly_stats'][year]

docs = data['documents']

cites = data['citations']

total_docs += docs

total_cites += cites

f.write(f"{year:<10} {docs:<15} {cites:<15}\n")

f.write("-" * 60 + "\n")

f.write(f"{'Total':<10} {total_docs:<15} {total_cites:<15}\n")

f.write("\n" + "=" * 60 + "\n")

f.write("合并策略说明:\n")

f.write("- WOS记录优先保留（数据更完整）\n")

f.write("- Scopus信息用于补充WOS缺失字段\n")

f.write("- 被引次数（TC）取两者最大值\n")

f.write("- Scopus独有记录（无WOS对应）已全部保留\n")

logger.info(f"\n详细报告已保存: {report_file}")

def main():

"""主函数"""

import sys

import argparse

\# 命令行参数解析

parser = argparse.ArgumentParser(

description='WOS和Scopus文献数据合并去重工具',

epilog='示例: python3 merge_deduplicate.py wos.txt scopus_converted.txt merged.txt'

)

parser.add_argument('wos_file', nargs='?', default='wos.txt',

help='WOS文件路径（默认: wos.txt）')

parser.add_argument('scopus_file', nargs='?', default='scopus_converted_to_wos.txt',

help='Scopus转换后的文件路径（默认: scopus_converted_to_wos.txt）')

parser.add_argument('output_file', nargs='?', default='merged_deduplicated.txt',

help='输出文件路径（默认: merged_deduplicated.txt）')

parser.add_argument('--log-level', choices=['DEBUG', 'INFO', 'WARNING', 'ERROR'],

default='INFO', help='日志级别（默认: INFO）')

args = parser.parse_args()

\# 设置日志级别

logging.getLogger().setLevel(getattr(logging, args.log_level))

try:

\# 执行合并去重

tool = MergeDeduplicateTool(args.wos_file, args.scopus_file, args.output_file)

tool.run()

return 0

except FileNotFoundError as e:

logger.error(f"文件错误: {e}")

return 1

except Exception as e:

logger.error(f"发生未知错误: {e}")

logger.exception("详细错误信息:")

return 1

if __name__ == '__main__':

main()
