# Supplementary material for: Infection-related research in Parkinson’s disease: an exploratory trends analysis
Source: Front Aging Neurosci. 2026 Oct 16;18:1959926. doi: 10.3389/fnagi.2026.1959926 (PMC13725743; doi:10.3389/fnagi.2026.1959926)
Supplement: Supplementary file 1 [file Data_Sheet_1.ZIP › supplementary data 2.docx]

VOSviewer was used to perform multidimensional bibliometric visualization. The minimum publication thresholds were set at 5 for countries/regions, 7 for institutions, 3 for authors, and 4 for source journals, yielding 50, 72, 74, and 86 nodes, respectively. For co-citation analyses, the minimum citation thresholds were 60 for journals and 20 for references, resulting in 267 co-cited journals and 151 co-cited references. In the keyword co-occurrence analysis,197 of 6,551 keywords met the minimum occurrence threshold of 10 and were included in the clustering analysis.
