# Supplementary material for: Infection-related research in Parkinson’s disease: an exploratory trends analysis
Source: Front Aging Neurosci. 2026 Oct 16;18:1959926. doi: 10.3389/fnagi.2026.1959926 (PMC13725743; doi:10.3389/fnagi.2026.1959926)
Supplement: Supplementary file 1 [file Data_Sheet_1.ZIP › supplementary data 3.docx]

| label | replace by |
| --- | --- |
| alzheimer disease | alzheimers disease |
| alzheimers diease | alzheimers disease |
| alzheimers disease (ad) | alzheimers disease |
| parkinsons-disease | parkinson's disease |
| alzheimers-disease | alzheimer's disease |
| alzheimers | alzheimer's disease |
| alzheimers-disease | alzheimer's disease |
| blood-brain barrier | blood brain barrier |
| blood-brain-barrier | blood brain barrier |
| central-nervous-system | central nervous system |
| cns | central nervous system |
| complication | complications |
| deep brain-stimulation | deep brain stimulation |
| deep-brain-stimulation | deep brain stimulation |
| dbs | deep brain stimulation |
| helicobacter-pylori infection | helicobacter pylori |
| idiopathic parkinsonism | parkinson's disease |
| movement-disorders | movement disorders |
| neurodegenerative disease | neurodegenerative diseases |
| neurodegenerative disorders | neurodegenerative diseases |
| neurological disorders | neurodegenerative diseases |
| risk-factor | risk factor |
| risk-factors | risk factor |
| virus-infection | viral infection |
| amyotrophic-lateral-sclerosis | amyotrophic lateral sclerosis |
| s disease | parkinson's disease |
| parkinsons disease (pd) | parkinson's disease |
| parkinson?s disease | parkinson's disease |
| parkinsonism | parkinson's disease |
| infection | infections |
| microbiome | microbiota |
| gut microbiome | gut microbiota |
| viral-infection | viral infection |
| multiple-sclerosis | multiple sclerosis |
| metaanalysis | meta-analysis |
| traumatic brain-injury | traumatic brain injury |
| substantia-nigra | substantia nigra |
| l-dopa | levodopa |
