# Supplementary material for: Infection-related research in Parkinson’s disease: an exploratory trends analysis
Source: Front Aging Neurosci. 2026 Oct 16;18:1959926. doi: 10.3389/fnagi.2026.1959926 (PMC13725743; doi:10.3389/fnagi.2026.1959926)
Supplement: Supplementary file 1 [file Data_Sheet_1.ZIP › supplementary data 4.docx]

| label | cluster | Occurrences |
| --- | --- | --- |
| accuracy | 1 | 15 |
| age | 1 | 13 |
| aids | 1 | 12 |
| antipsychotics | 1 | 11 |
| ataxia | 1 | 13 |
| basal ganglia | 1 | 31 |
| burden | 1 | 17 |
| care | 1 | 12 |
| chorea | 1 | 10 |
| clinical-features | 1 | 10 |
| clozapine | 1 | 11 |
| cohort | 1 | 11 |
| comorbidity | 1 | 12 |
| complications | 1 | 83 |
| death | 1 | 18 |
| deep brain stimulation | 1 | 144 |
| delirium | 1 | 10 |
| dementia | 1 | 77 |
| depression | 1 | 29 |
| diagnosis | 1 | 51 |
| diagnostic-criteria | 1 | 10 |
| disease | 1 | 98 |
| disorders | 1 | 25 |
| double-blind | 1 | 34 |
| dysfunction | 1 | 32 |
| dysphagia | 1 | 17 |
| dystonia | 1 | 38 |
| efficacy | 1 | 26 |
| epidemiology | 1 | 30 |
| essential tremor | 1 | 22 |
| follow-up | 1 | 21 |
| globus-pallidus | 1 | 24 |
| hardware-related complications | 1 | 23 |
| health | 1 | 20 |
| hiv | 1 | 19 |
| hospitalization | 1 | 17 |
| impact | 1 | 25 |
| impairment | 1 | 14 |
| implantation | 1 | 11 |
| involvement | 1 | 13 |
| management | 1 | 48 |
| medical therapy | 1 | 17 |
| meta-analysis | 1 | 58 |
| morbidity | 1 | 10 |
| mortality | 1 | 59 |
| motor | 1 | 13 |
| movement disorders | 1 | 71 |
| multiple system atrophy | 1 | 25 |
| nonmotor symptoms | 1 | 18 |
| nucleus | 1 | 11 |
| outcm | 1 | 36 |
| pain | 1 | 11 |
| patient | 1 | 15 |
| people | 1 | 20 |
| pneumonia | 1 | 11 |
| population | 1 | 13 |
| prevalence | 1 | 57 |
| progression | 1 | 27 |
| progressive supranuclear palsy | 1 | 18 |
| psychosis | 1 | 11 |
| quality | 1 | 10 |
| quality-of-life | 1 | 25 |
| risk | 1 | 107 |
| risk factor | 1 | 61 |
| safety | 1 | 23 |
| stress | 1 | 15 |
| subthalamic nucleus | 1 | 66 |
| subthalamic nucleus stimulation | 1 | 15 |
| surgery | 1 | 69 |
| survival | 1 | 18 |
| symptoms | 1 | 31 |
| systematic review | 1 | 16 |
| telemedicine | 1 | 12 |
| term | 1 | 10 |
| therapy | 1 | 37 |
| tremor | 1 | 26 |
| trial | 1 | 16 |
| urinary tract infection | 1 | 11 |
| validation | 1 | 12 |
| a-beta | 2 | 13 |
| activation | 2 | 43 |
| aggregation | 2 | 10 |
| aging | 2 | 15 |
| alpha-synuclein | 2 | 165 |
| alzheimer's disease | 2 | 224 |
| amyloid-beta | 2 | 18 |
| amyotrophic lateral sclerosis | 2 | 44 |
| apoptosis | 2 | 11 |
| autophagy | 2 | 14 |
| biomarker | 2 | 19 |
| cancer | 2 | 33 |
| case report | 2 | 15 |
| cell death | 2 | 15 |
| cells | 2 | 42 |
| creutzfeldt-jakob-disease | 2 | 19 |
| expression | 2 | 64 |
| gene | 2 | 24 |
| genome-wide association | 2 | 10 |
| herpes-simplex-virus | 2 | 11 |
| huntingtons-disease | 2 | 10 |
| identification | 2 | 16 |
| in-vitro | 2 | 26 |
| in-vivo | 2 | 17 |
| influenza-a virus | 2 | 11 |
| inhibition | 2 | 17 |
| ischemic stroke | 2 | 32 |
| lewy bodies | 2 | 35 |
| lrrk2 | 2 | 21 |
| mechanism | 2 | 37 |
| mitochondrial dysfunction | 2 | 22 |
| model | 2 | 52 |
| mutation | 2 | 25 |
| neurodegenerative diseases | 2 | 109 |
| oxidative stress | 2 | 63 |
| parkinson | 2 | 27 |
| pathology | 2 | 28 |
| pathway | 2 | 13 |
| prion | 2 | 20 |
| prion protein | 2 | 11 |
| protein | 2 | 48 |
| protein aggregation | 2 | 10 |
| tau | 2 | 11 |
| transmission | 2 | 18 |
| astrocytes | 3 | 21 |
| blood brain barrier | 3 | 29 |
| brain | 3 | 93 |
| central nervous system | 3 | 113 |
| cerebrospinal-fluid | 3 | 53 |
| cytokines | 3 | 25 |
| dopamine | 3 | 43 |
| dopaminergic-neurons | 3 | 28 |
| epilepsy | 3 | 14 |
| inflammatory responses | 3 | 105 |
| innate immunity | 3 | 16 |
| lipopolysaccharide | 3 | 10 |
| microglia cell | 3 | 73 |
| microglial activation | 3 | 25 |
| mitochondria | 3 | 22 |
| mouse model | 3 | 48 |
| mptp | 3 | 10 |
| multiple sclerosis | 3 | 71 |
| necrosis-factor-alpha | 3 | 19 |
| nervous-system | 3 | 20 |
| neurodegeneration | 3 | 121 |
| neuroinflammation | 3 | 126 |
| neuroprotection | 3 | 15 |
| neurotoxicity | 3 | 15 |
| neurotrophic factor | 3 | 12 |
| nf-kappa-b | 3 | 30 |
| nitric oxide | 3 | 24 |
| nlrp3 inflammasome | 3 | 20 |
| nonsteroidal antiinflammatory drugs | 3 | 10 |
| protect | 3 | 12 |
| rat model | 3 | 10 |
| substantia nigra | 3 | 74 |
| t-cells | 3 | 19 |
| toll-like receptor | 3 | 14 |
| toxicity | 3 | 11 |
| traumatic brain injury | 3 | 19 |
| tumor-necrosis-factor | 3 | 16 |
| viral infection | 3 | 32 |
| amantadine | 4 | 16 |
| antibody | 4 | 20 |
| association | 4 | 65 |
| bacteria | 4 | 13 |
| c virus-infection | 4 | 11 |
| cognitive impairment | 4 | 24 |
| coronavirus | 4 | 43 |
| covid-19 | 4 | 179 |
| encephalitis | 4 | 35 |
| encephalitis lethargica | 4 | 17 |
| etiology | 4 | 19 |
| exposure | 4 | 14 |
| h5n1 influenza-virus | 4 | 15 |
| influenza | 4 | 19 |
| mice | 4 | 31 |
| neurons | 4 | 27 |
| postencephalitic parkinsonism | 4 | 12 |
| receptor | 4 | 20 |
| sars-cov-2 | 4 | 98 |
| schizophrenia | 4 | 11 |
| system | 4 | 19 |
| toxoplasma gondii | 4 | 10 |
| virus | 4 | 42 |
| dysbiosis | 5 | 13 |
| eradication | 5 | 25 |
| gastrointestinal-tract | 5 | 14 |
| gut microbiota | 5 | 48 |
| gut-brain axis | 5 | 28 |
| helicobacter pylori | 5 | 63 |
| intestinal bacterial overgrowth | 5 | 14 |
| l-dopa absorption | 5 | 14 |
| levodopa | 5 | 67 |
| manifestations | 5 | 10 |
| microbiota | 5 | 25 |
| motor fluctuations | 5 | 29 |
| pathogenesis | 5 | 46 |
